# Supplementary material for: A dual spin-controlled chiral two-/three-dimensional perovskite artificial leaf for efficient overall photoelectrochemical water splitting
Source: Nat Commun. 2024 Jun 1;15:4672. doi: 10.1038/s41467-024-49216-x (PMC11144254; doi:10.1038/s41467-024-49216-x)
Supplement: Supplementary file 1 — Supplementary Information [file 41467_2024_49216_MOESM1_ESM.pdf]

Supplementary Information for

A Dual Spin-Controlled Chiral Two-/Three-Dimensional Perovskite Artificial Leaf for Efficient Overall Photoelectrochemical Water Splitting

*Hyungsoo Lee†, Chan Uk Lee†, Juwon Yun, Chang-Seop Jeong, Wooyong Jeong, Jaehyun Son, Young Sun Park, Subin Moon, Soobin Lee, Jun Hwan Kim, and Jooho Moon**

*Email: jmoon@yonsei.ac.kr;

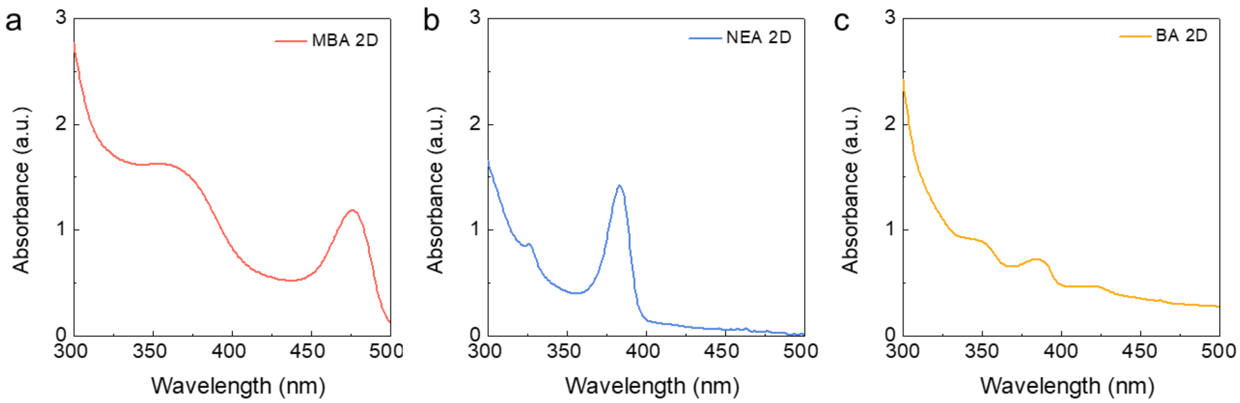

Supplementary Fig. S1. Absorbance spectra of perovskites (a) $(MBA\text{-}2D)_2PbBr_4$, (b) $(NEA\text{-}2D)_2PbBr_4$, and (c) $(BA\text{-}2D)_2PbBr_4$ on FTO substrate. MBA, α-methylbenzylamine; NEA, 1-(2-naphthyl) ethylamine; BA, *sec*-butylamine.

Supplementary Note 1. Measuring the anisotropy of the spin-dependent current via magnetic conducting probe atomic force microscopy (mCP-AFM).

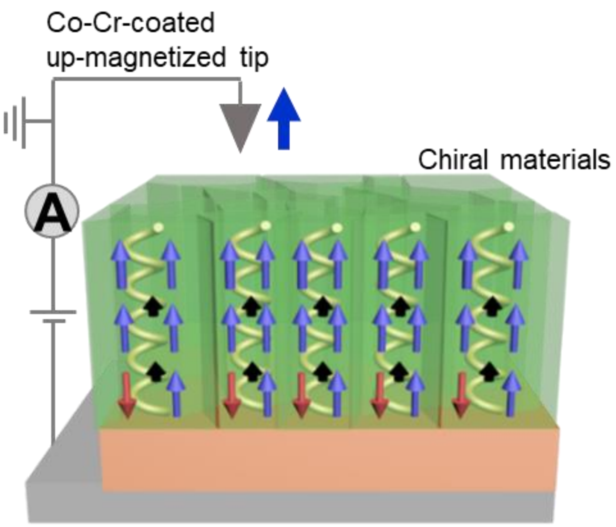

Supplementary Fig. S2. A schematic illustration of mCP-AFM measurements under an up-magnetized field for spin polarizability calculations.

As adapted from a previous report,[R1] the spin-dependent current developed by the 2D chiral lead halide hybrid perovskites (OIHPs) was measured using mCP-AFM with a pre-magnetized Co-Cr-coated tip (Supplementary Fig. S2). The anisotropy of the spin-dependent current represents the spin-polarization degree (P_V), defined as follows:

$$P_V = \frac{I_{down}-I_{up}}{I_{down}+I_{up}} \times 100\%, \tag{S1}$$

where I_{down} and I_{up} are the measured spin-dependent currents at specific voltage V when the tip is pre-magnetized with down and up field orientations, respectively. The calculated spin-polarization degree by the 2D chiral OIHP thin films clearly demonstrates their spin-dependent current behavior according to the handedness of the chiral organic molecule.

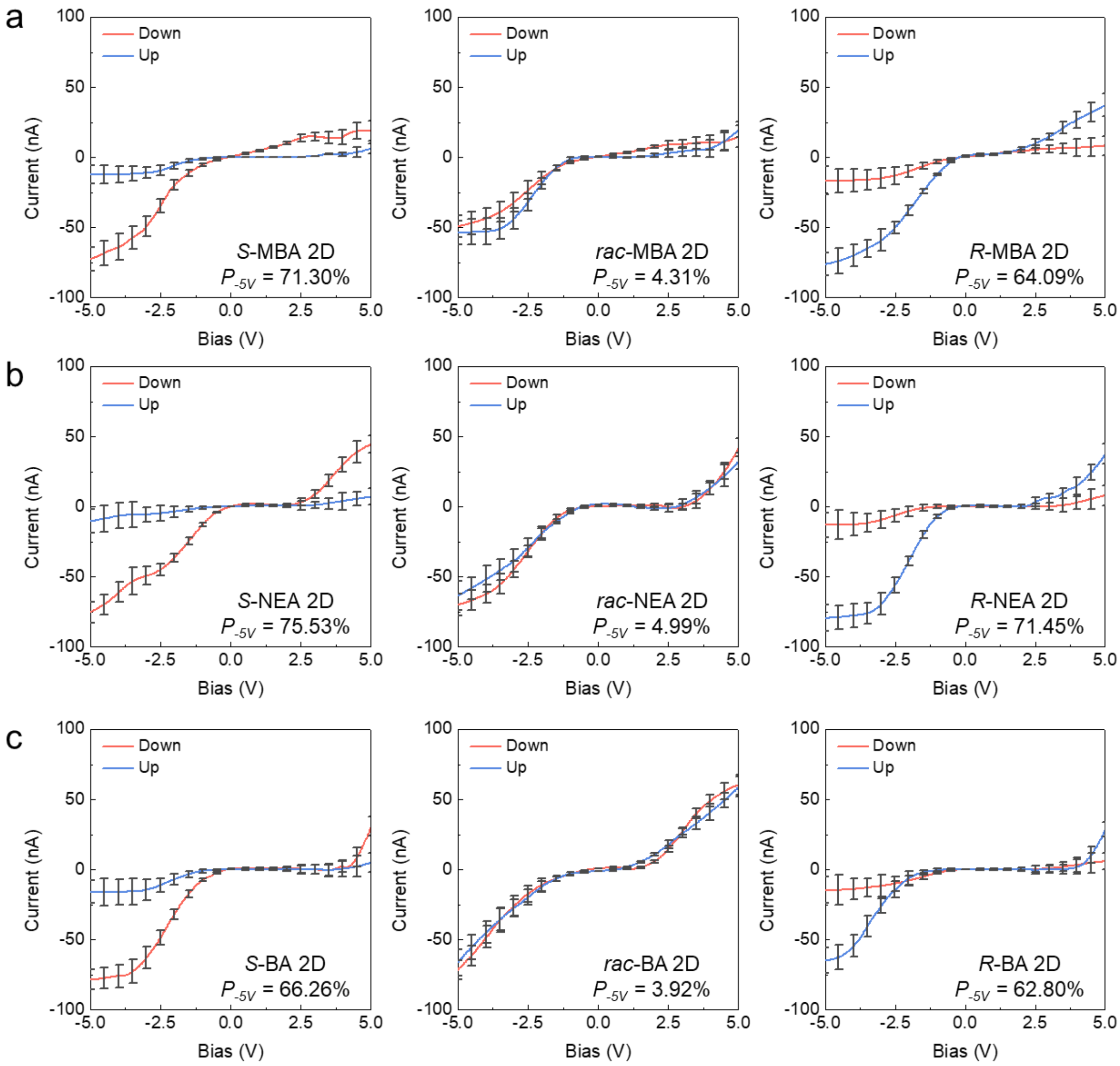

Supplementary Fig. S3. Spin-dependent current values for the (a) MBA-, (b) NEA-, and (c) BA-based 2D chiral OIHPs coated on fluorine-doped tin oxide (FTO) substrates depending on the chirality (*S-/rac-/R*-configuration) measured using mCP-AFM with a pre-magnetized Co-Cr-coated tip.

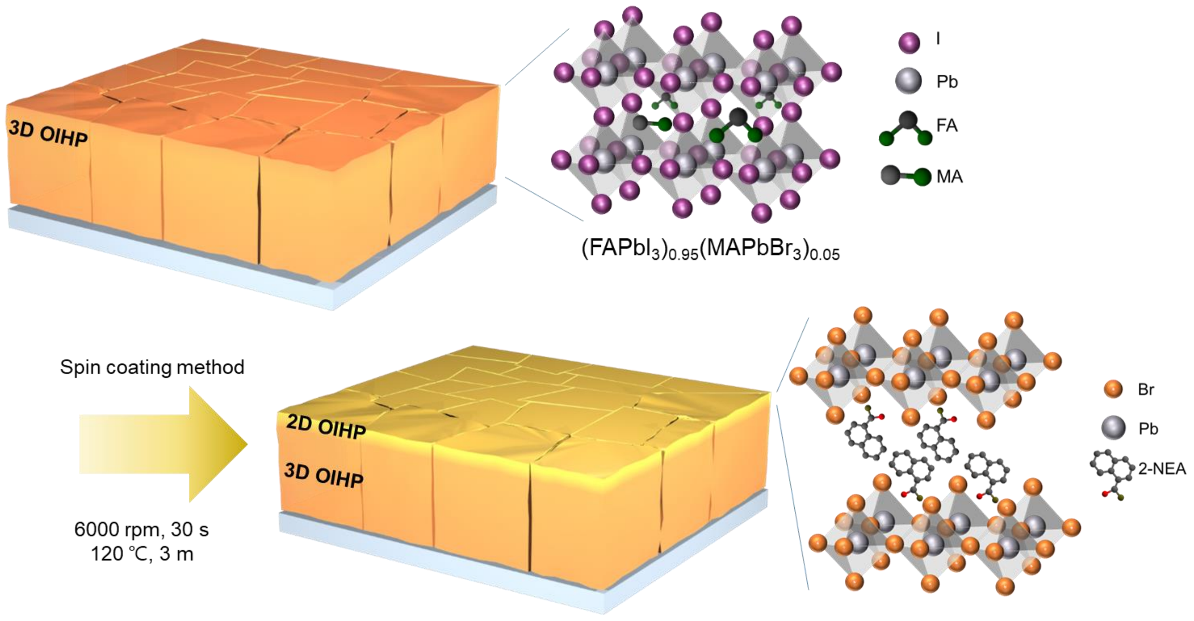

Supplementary Fig. S4. A schematic of the fabrication of the ultra-thin 2D chiral 2-NEA_2PbBr_4 films (NEA 2D OIHP) on top of a 3D $(FAPbI_3)_{0.95}(MAPbBr_3)_{0.05}$ film. The NEA-Br chiral cation salt-based molecular ink was spin-coated onto the 3D OIHP layer at 6000 rpm for 30 s. Subsequent annealing at 120 ℃ for 3 m enabled the formation of the NEA-based 2D chiral OIHP.

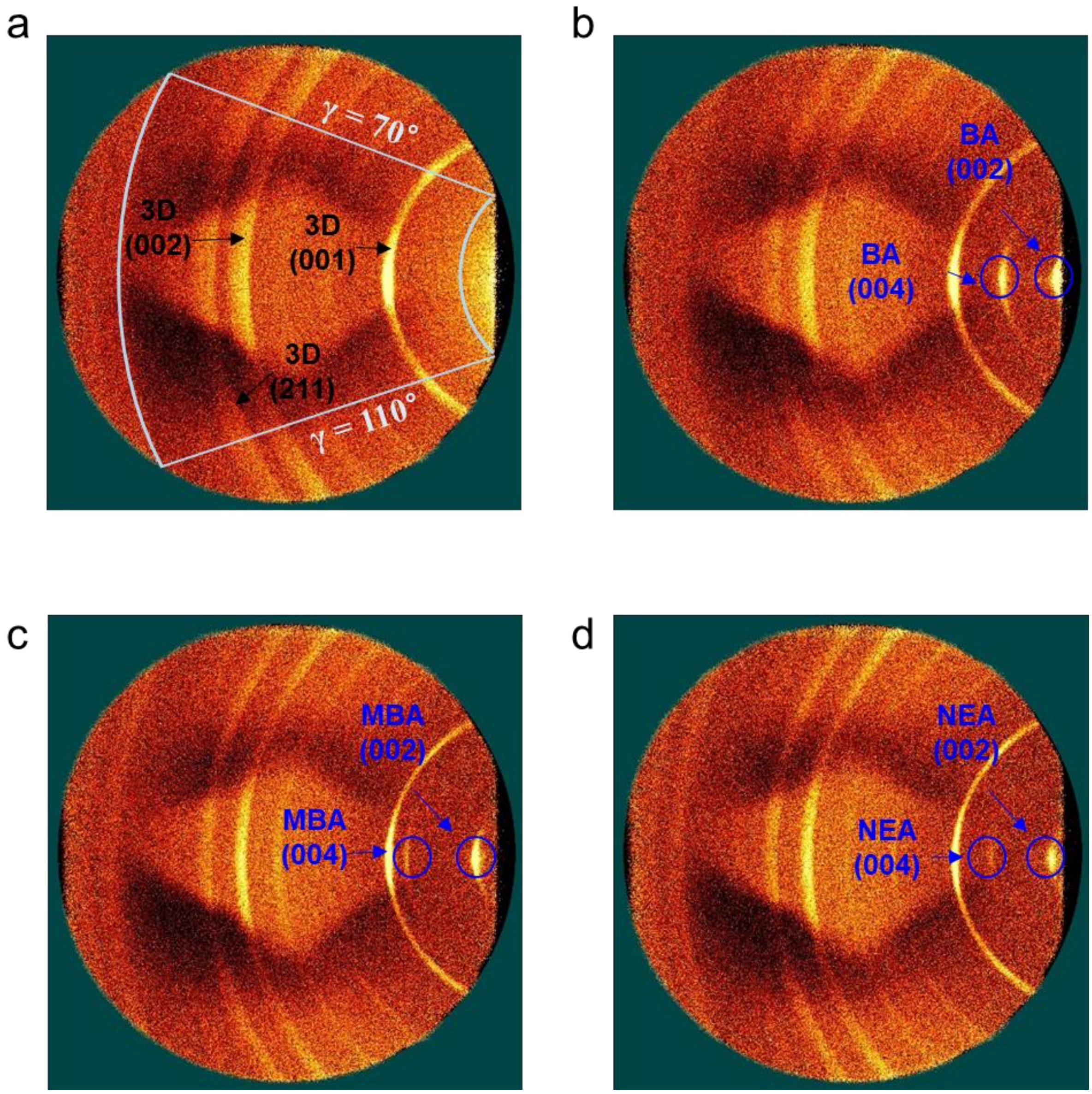

Supplementary Fig. S5. 2D X-ray diffraction spectra with general area detector diffraction system (2D GADDS XRD) of (a) 3D OIHP, (b) BA 2D/3D OIHP, (c) MBA 2D/3D OIHP, and (d) NEA 2D/3D OIHP on FTO substrates conducted with $70° < \gamma < 110°$.

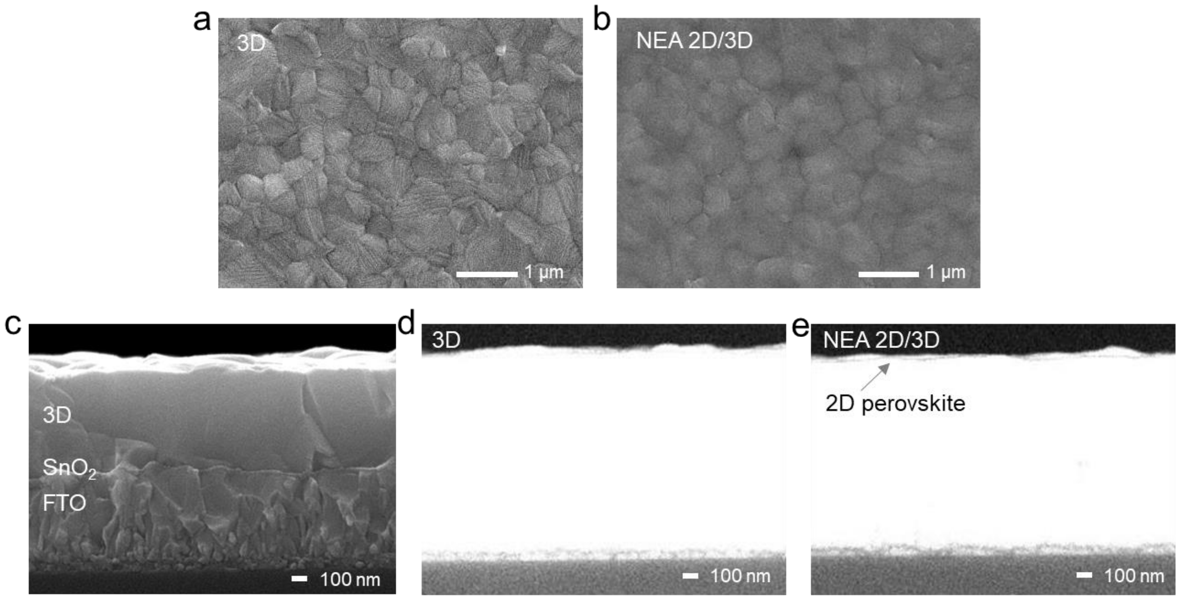

Supplementary Fig. S6. Surface scanning electron microscopy (SEM) images of (a) 3D OIHP and (b) NEA 2D/3D OIHP on a silicon dioxide (SnO_2)/FTO substrate. The cross-sectional SEM images of (c) 3D OIHP on a SnO_2/FTO substrate. The cross-sectional backscattered SEM images (COMPO mode) of (d) 3D OIHP and (e) NEA 2D/3D OIHP on a SnO_2/FTO substrate.

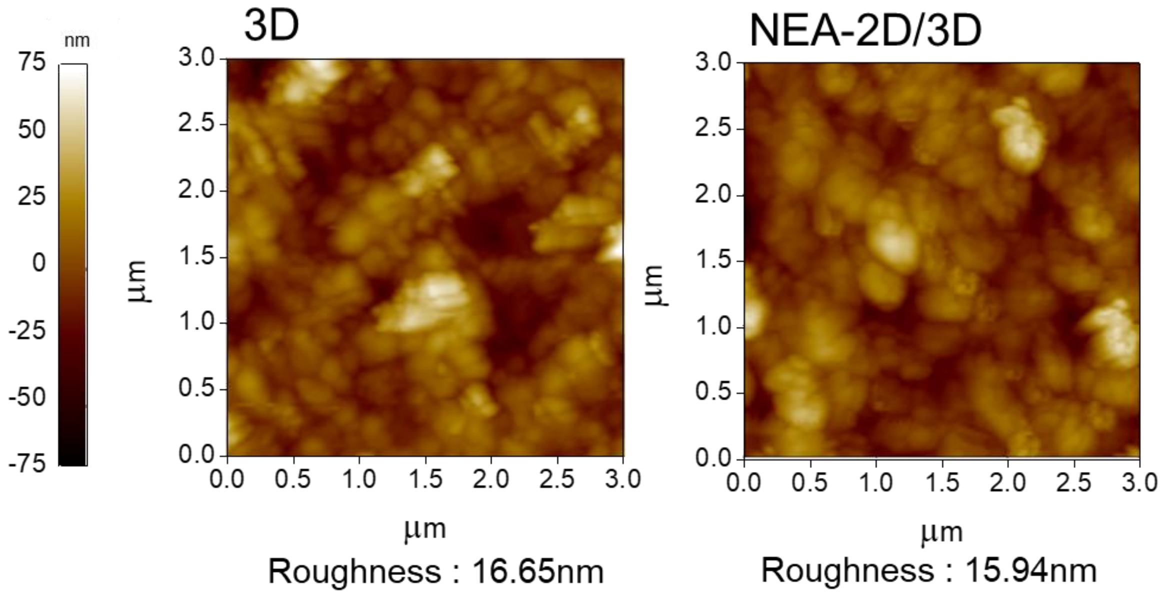

Supplementary Fig. S7. Topography and surface roughness of the 3D OIHP and NEA-based 2D/3D OIHP films obtained via AFM measurements.

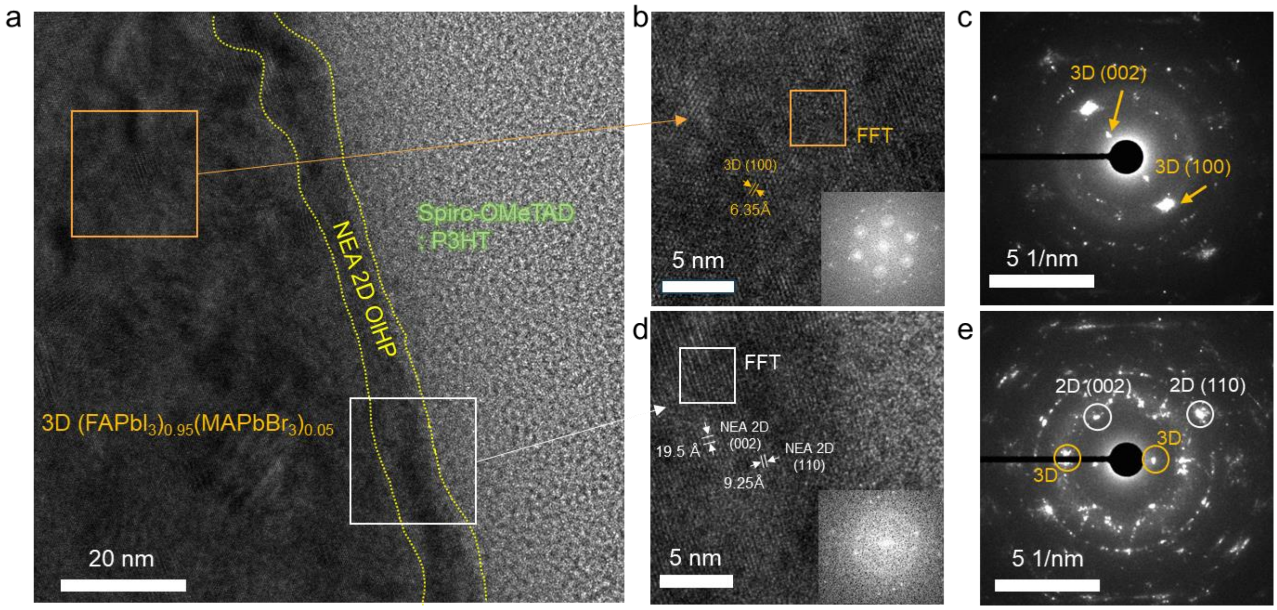

Supplementary Fig. S8. (a) A cross-sectional transmission electron microscopy (TEM) image of the 88 vol% spiro-MeOTAD:12 vol% P3HT (poly(3-hexylthiophene-2,5-diyl) hole transport layer (HTL)/2D NEA_2PbBr_4/3D $(FAPbI_3)_{0.95}(MAPbBr_3)_{0.05}$ structure. (b) A high-resolution (HR)-TEM image of the orange box in (a) of the 3D OIHP with the lattice fringe and diffraction pattern enhanced via fast Fourier-transform (FFT) and (c) the corresponding selected area electron diffraction (SAED) pattern showing the reciprocal lattices of the 3D OIHP. (d) An HR-TEM image of the HTL/NEA-2D/3D OIHP interface (white box) as well as the related lattice fringes and diffraction patterns enhanced via FFT and (e) a SAED pattern of the corresponding area involving the reciprocal lattices of the NEA 2D chiral OIHP and the 3D OIHP.

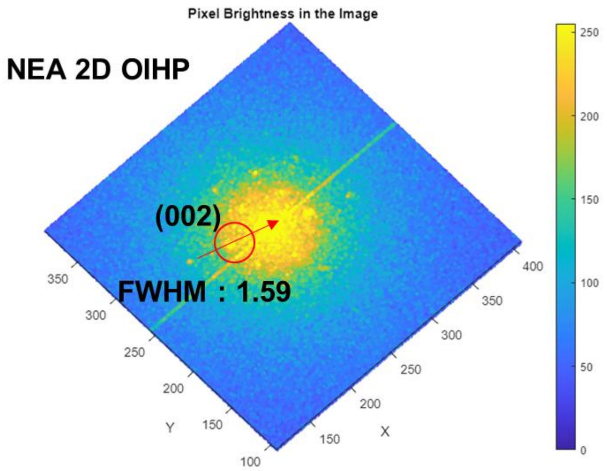

Supplementary Fig. S9. Calculation of the full-width half the maximum intensity (FWHM) of *S*-NEA 2D OIHP films from the FFT of white region in Supplementary Fig. S8d.

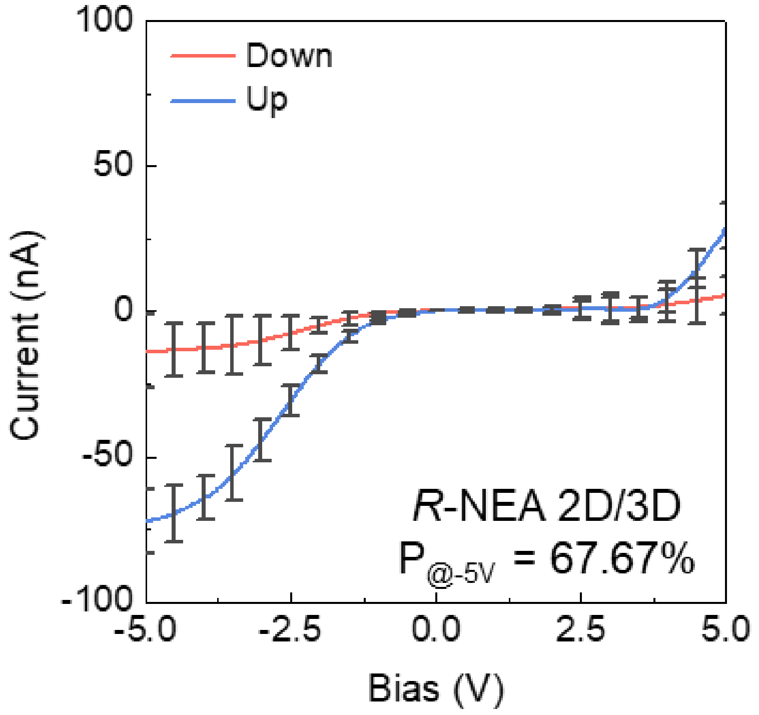

Supplementary Fig. S10. The spin-dependent current generated in the *R*-NEA 2D/3D/SnO_2/FTO structure measured by using mCP-AFM with a pre-magnetized Co-Cr-coated tip.

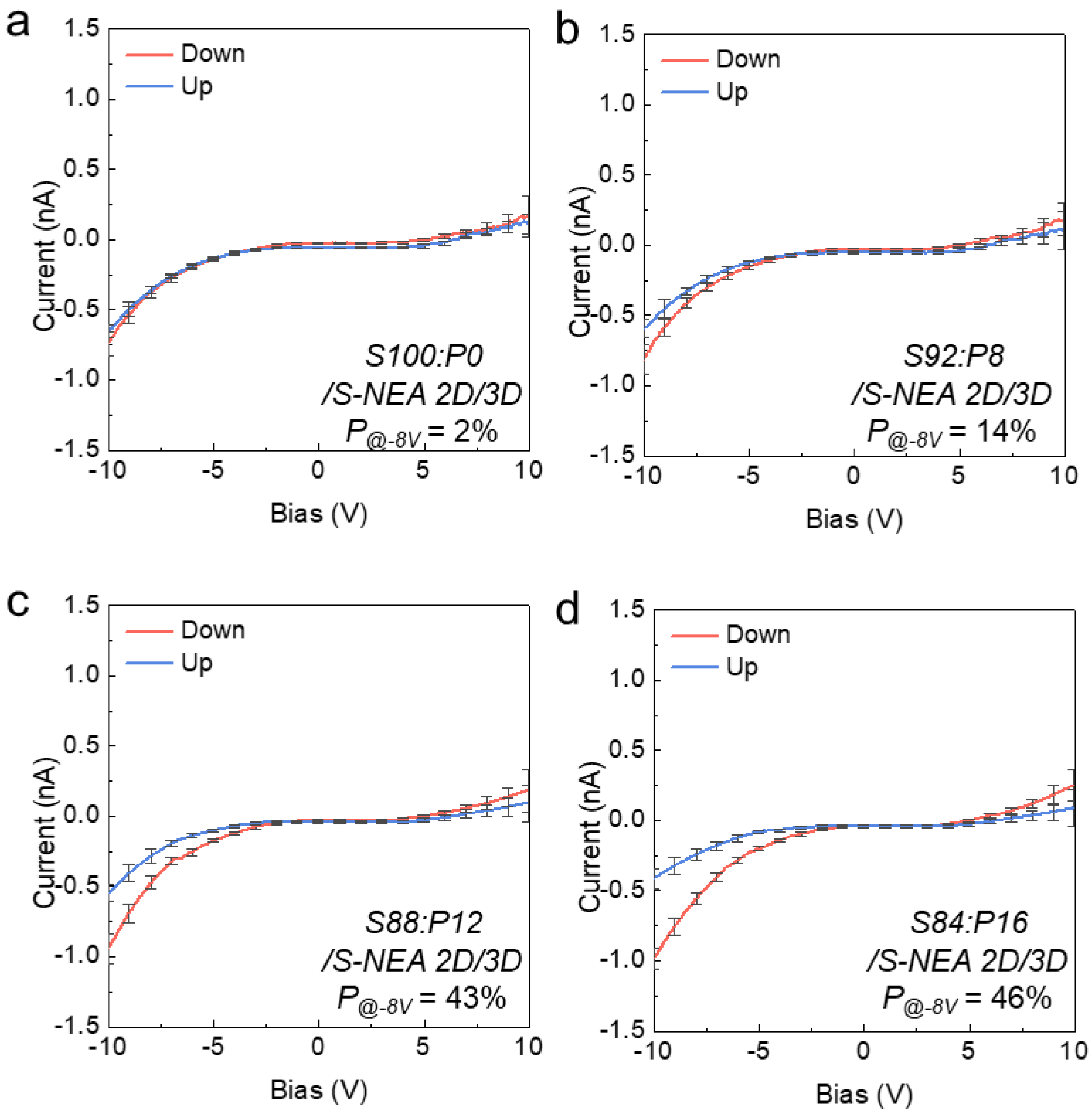

Supplementary Fig. S11. The spin-dependent current generated in the HTL/*S*-NEA 2D/3D/SnO_2/FTO device with an HTL comprising various volume ratios of spiro-MeOTAD:P3HT (i.e., (a) S100:P0, (b) S92:P8, (c) S88:P12, (d) S84:P16) measured using mCP-AFM with a pre-magnetized Co-Cr-coated tip.

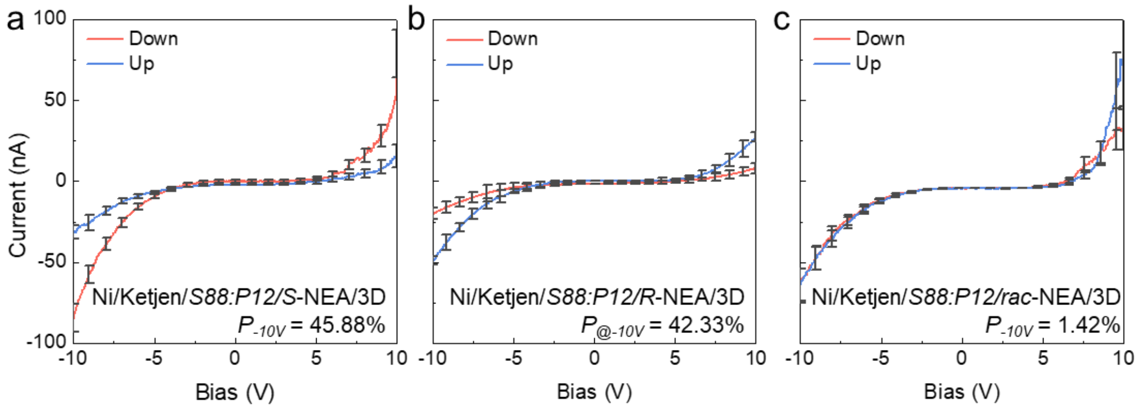

Supplementary Fig. S12. Spin-dependent current generated of Ni/Ketjen/S88:P12/NEA 2D/3D OIHP in which various configurations of NEA were utilized: (a) *S*-NEA 2D, (b) *R*-NEA 2D and (c) *rac*-NEA 2D. mCP-AFM was measured in the range from –10 to 10 V.

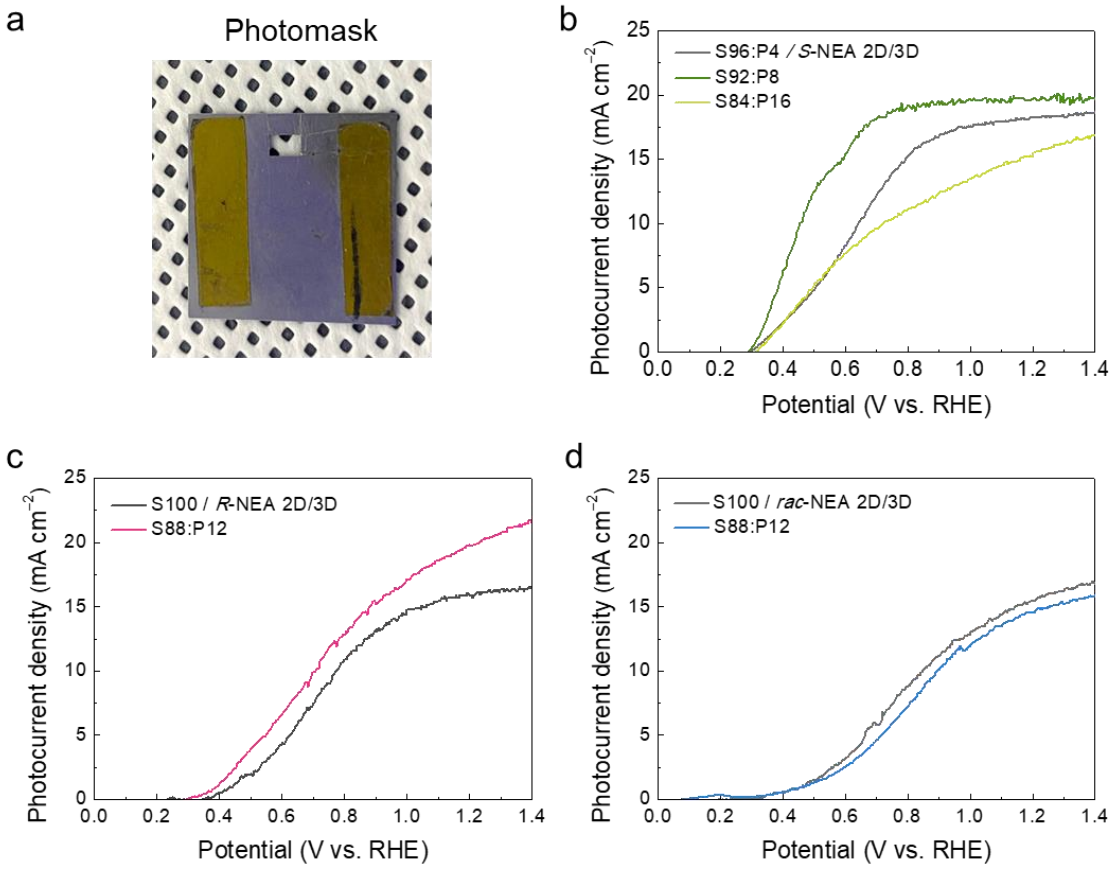

Supplementary Fig. S13. (a) Photograph of photomask with an area of 0.06 cm^2 to define the light absorption area. Linear sweep voltammetry (LSV) curves of (b) *S*-NEA 2D/3D OIHP, (c) *R*-NEA 2D/3D OIHP, and (d) *rac*-NEA 2D/3D OIHP photoanodes with various spiro-MeOTAD:P3HT volume ratios in a K-Pi electrolyte (pH 6.5) under 1 sun illumination.

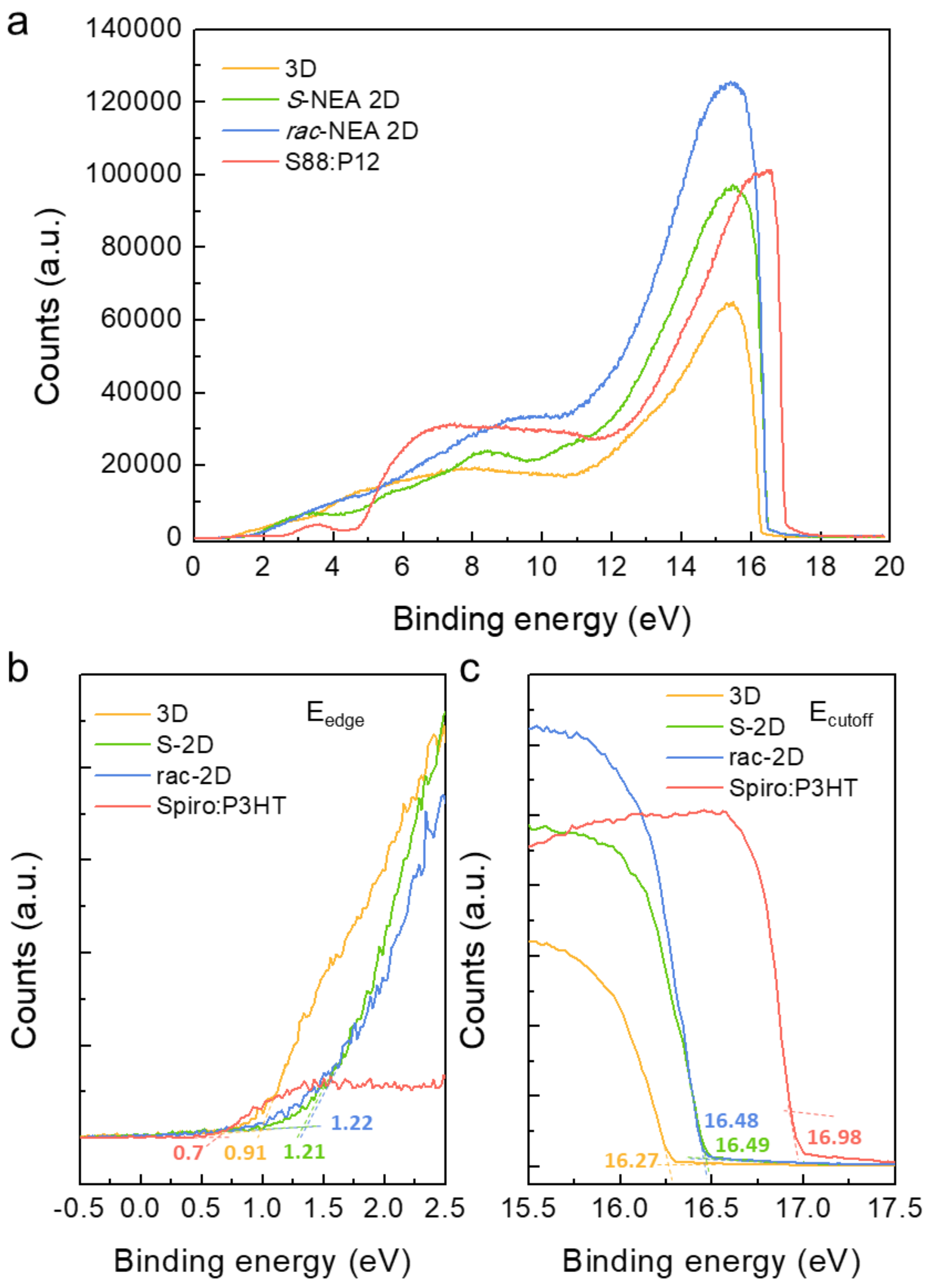

Supplementary Fig. S14. (a) Ultraviolet photoelectron spectra for the $(FAPbI_3)_{0.95}(MAPbBr_3)_{0.05}$ 3D OIHP, (*S*-/*rac*-NEA)$_2$PbBr$_4$ 2D OIHP, and spiro-MeOTAD:P3HT obtained using He I radiation at 21.21 eV. (b) E_{edge} and (c) E_{cutoff} to calculate the band energy position of valence band maximum and Fermi energy level.

Supplementary Note 2. Methodology of UPS spectra analysis.

Firstly, Supplementary Fig. S14b depicts the valence-band edge (E_{edge}) for each material, which represents the difference between valence band maximum (E_{VBM}) and Fermi energy level (E_F). Using the E_{edge} value, the definite VBM level of the sample was calculated as follows:

$$E_{VBM} = E_F - E_{edge} \qquad (S2)$$

The secondary-electron cutoff (E_{cutoff}) obtained via extrapolation to the linear part of the binding-energy edge is shown in Supplementary Fig. S14c. The E_F of each material was calculated using the following equation:

$$E_F = E_{cutoff} - 21.21 \text{ eV (under He I radiation)} \qquad (S3)$$

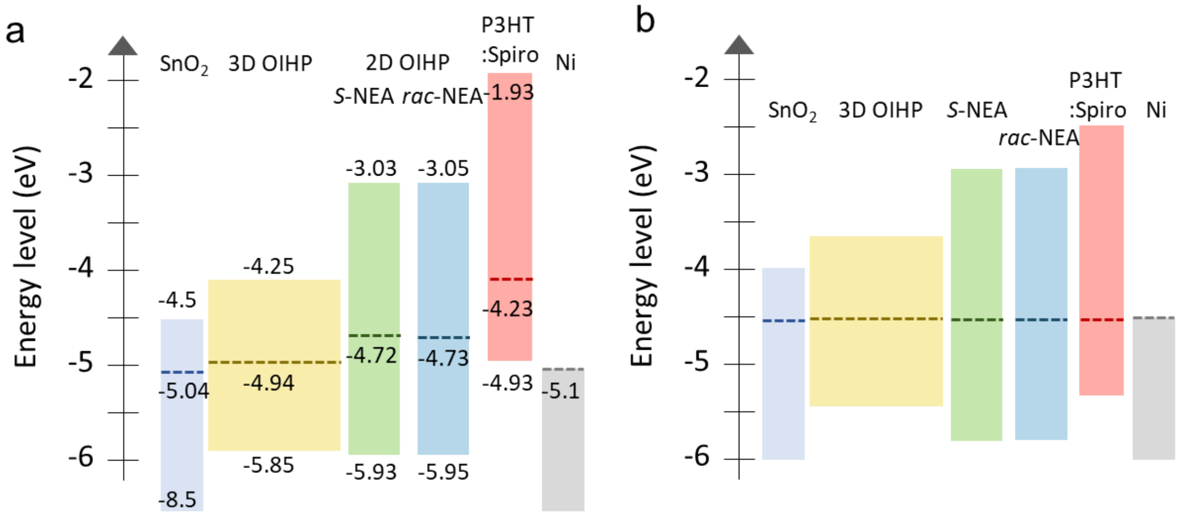

Supplementary Fig. S15. Band alignment of Ni/S88:P12/NEA 2D/3D/SnO_2 configuration for (a) pre-equilibrium state and (b) equilibrium state.

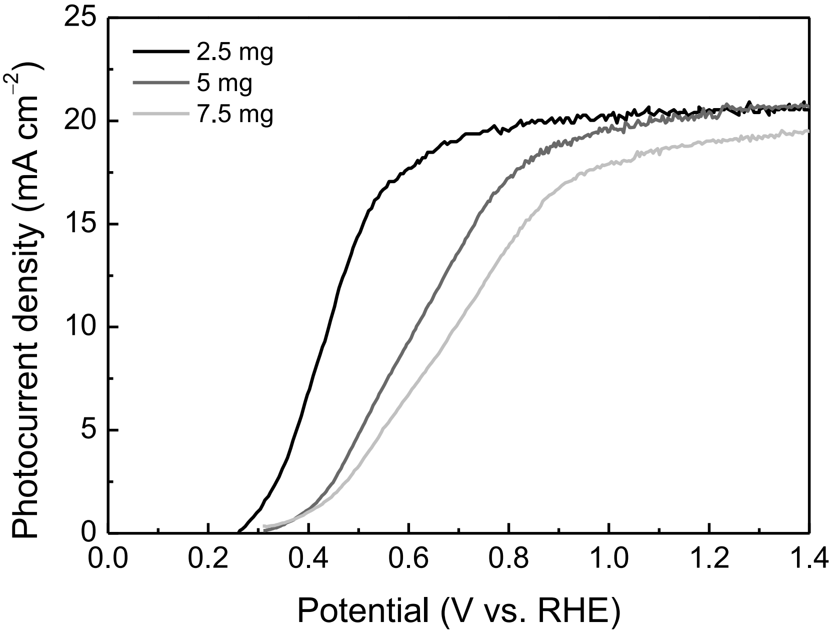

Supplementary Fig. S16. As the concentration of organic *S*-NEA cation increases (from 2.5 to 7.5 mg), it is possible to control the thickness of *S*-NEA layer on top of the 3D OHIP. LSV curves of *S*-NEA/3D photoanodes as a function of the thickness of *S*-NEA layer under 1 sun illumination in a K-Pi electrolyte (pH 6.5) with an area of 0.06 cm^2.

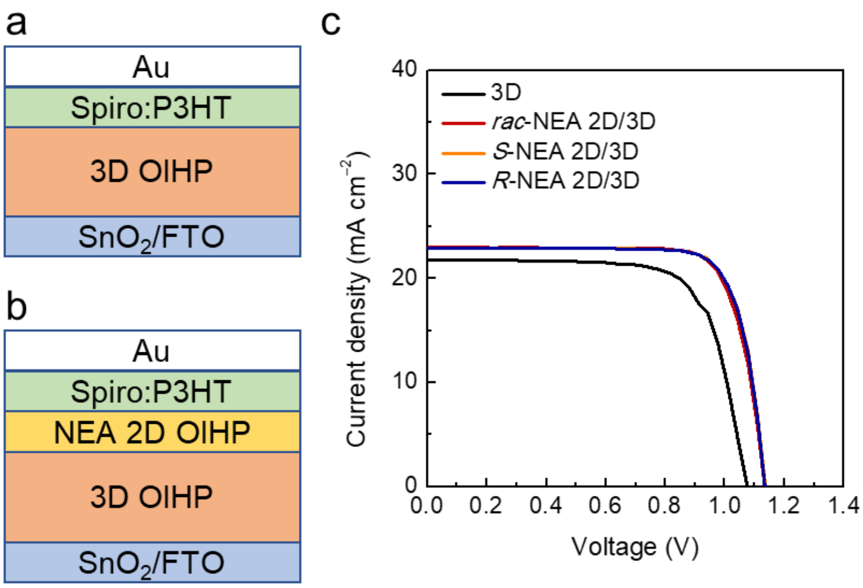

Supplementary Fig. S17. Schematic illustration of the 3D OIHP photovoltaic cells configuration (a) with or (b) without NEA 2D OIHP. (c) J–V curves and photovoltaic parameters of the champion PV cells based on the 3D, *rac*-NEA 2D/3D, *R*-NEA 2D/3D, and *S*-NEA 2D/3D OIHPs.

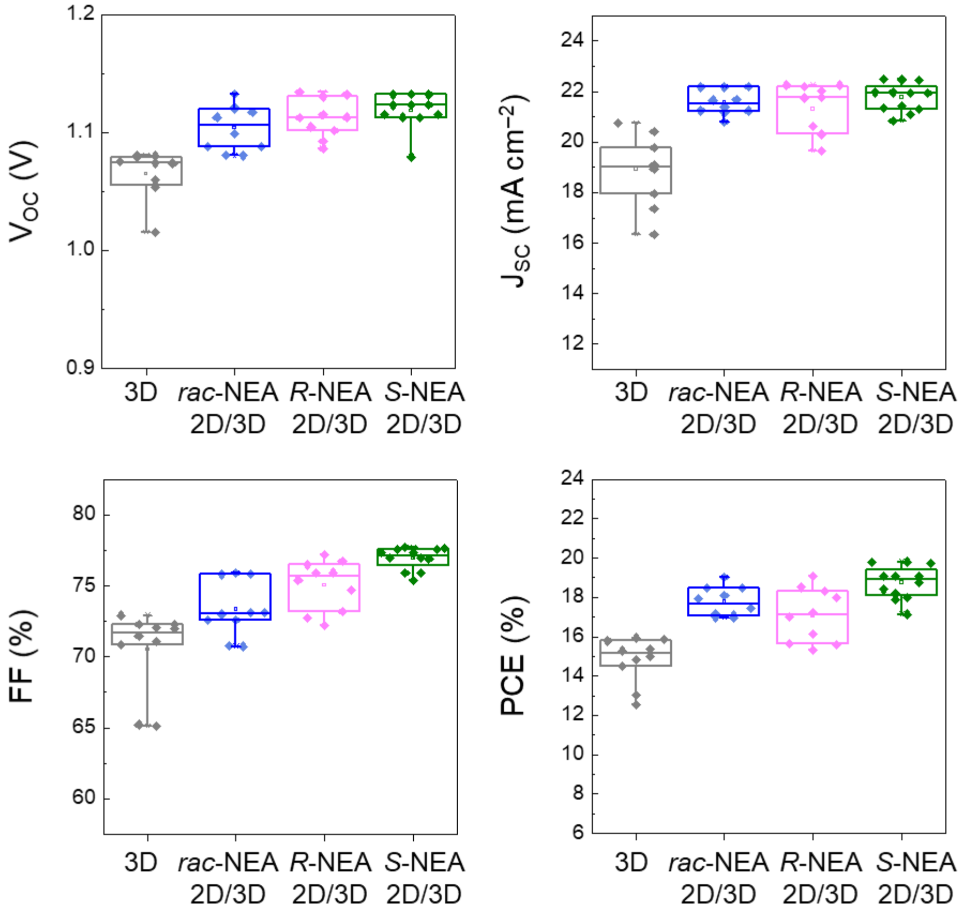

Supplementary Fig. S18. Statistical distribution of the photovoltaic parameters for the 3D, *rac*-NEA 2D/3D, *R*-NEA 2D/3D, and *S*-NEA 2D/3D based PV cells.

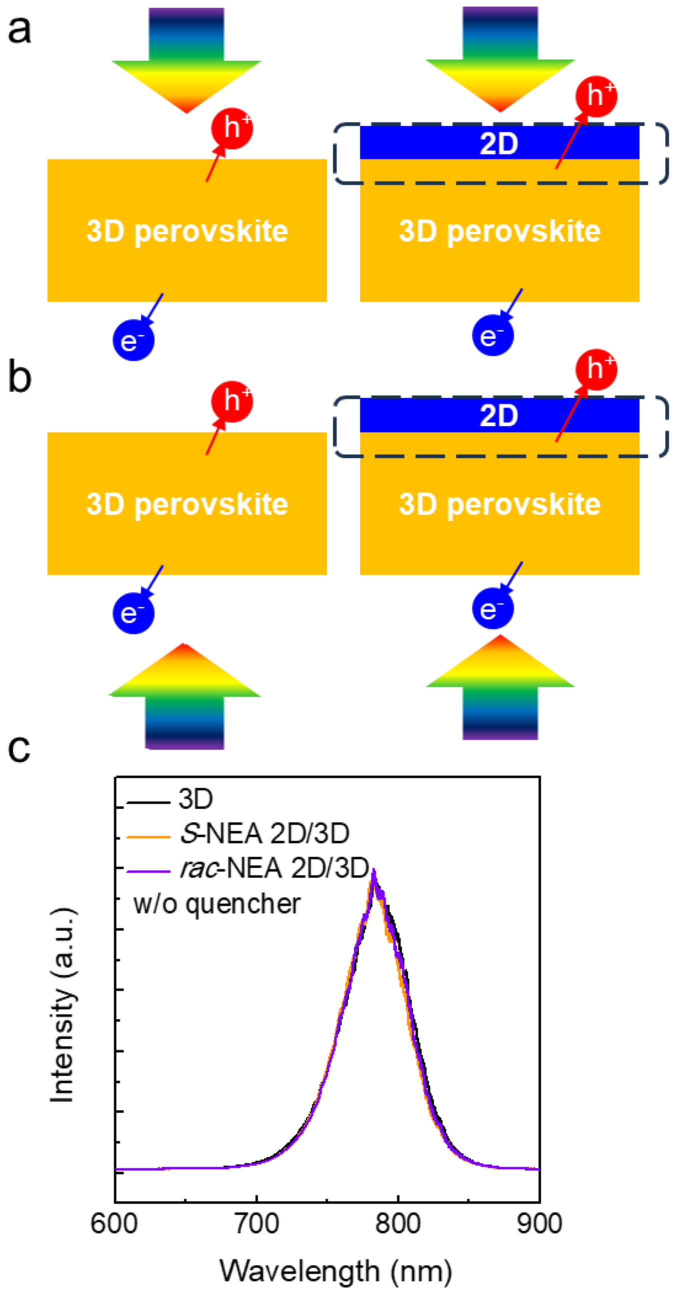

Supplementary Fig. S19. Illustrations of (a) front-excitation in which photon is injected into the NEA 2D/3D interface and (b) back-excitation which is an opposite illumination as compared to the front-excitation. (c) Steady-state photoluminescence (PL) measurements of 3D OIHP, *S*-NEA 2D/3D OIHP, and *rac*-NEA 2D/3D OIHP films on soda-lime glass (SLG) under back-excitation conditions without a quencher.

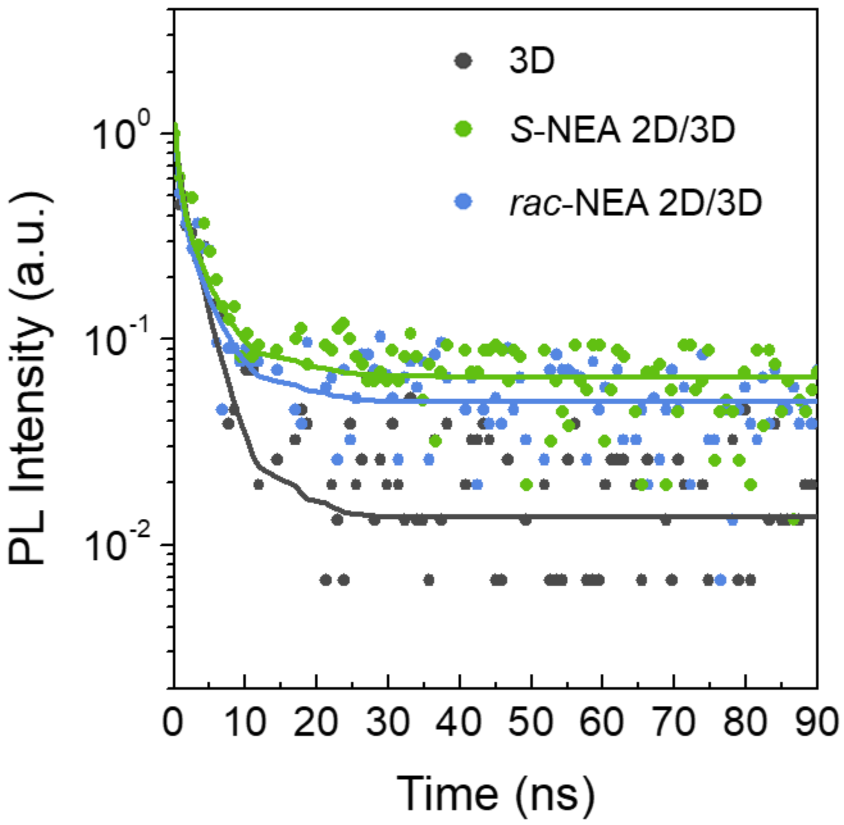

Supplementary Fig. S20. Time-resolved PL spectra of 3D, *S*-NEA 2D/3D, and *rac*-NEA 2D/3D films on soda-lime glass (SLG) measured without a quencher.

Supplementary Table. S1. TRPL fitting data of the fast (τ_1) and slow (τ_2) lifetime components and weight fraction (A) obtained using a biexponential model for the OIHP on the SLG samples.

	**A_1 (%)**	**τ_1 (ns)**	**A_2 (%)**	**τ_2 (ns)**
**3D**	36.93255	0.82	63.06745	3.33
***S*-NEA 2D/3D**	59.39655	0.83	40.60345	4.53
***rac*-NEA 2D/3D**	59.81247	0.83	40.18753	4.43

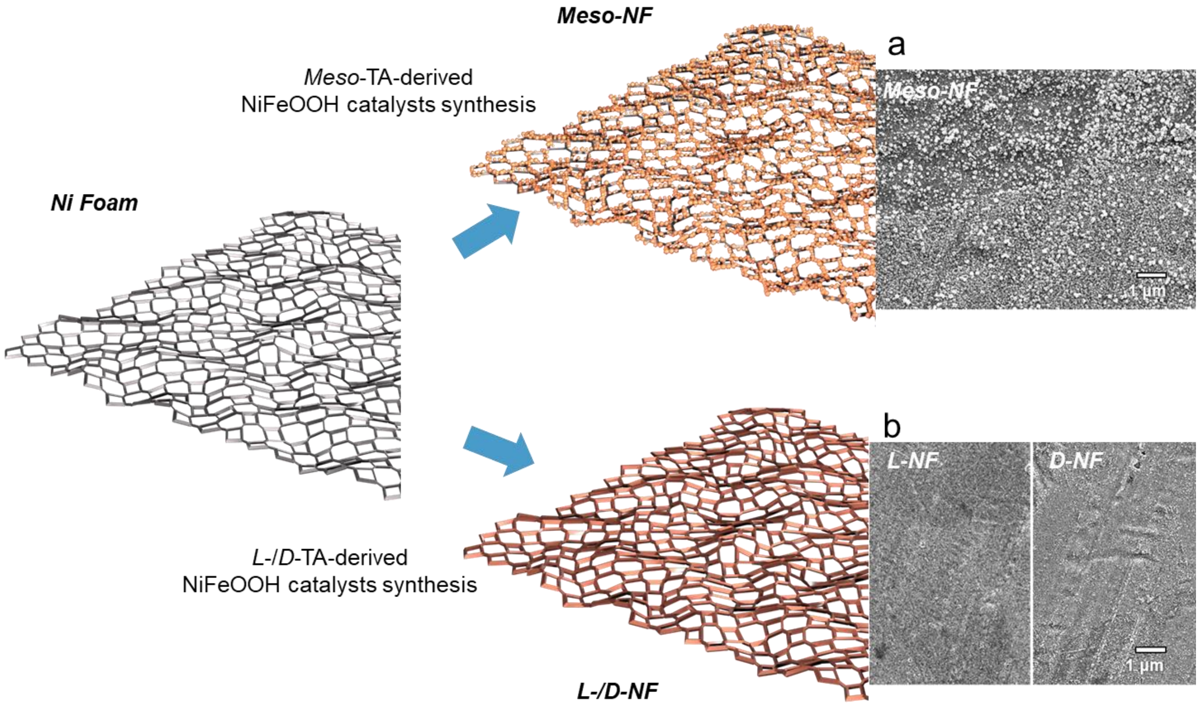

Supplementary Fig. S21. Enantioselective synthesis of the nickel-iron oxyhydroxide NiFeOOH (NF) catalysts using *meso*-/*L*-/*D*-TA and SEM images of (a) *meso*-NF and (b) *L*-/*D*-NF on Ni foam.

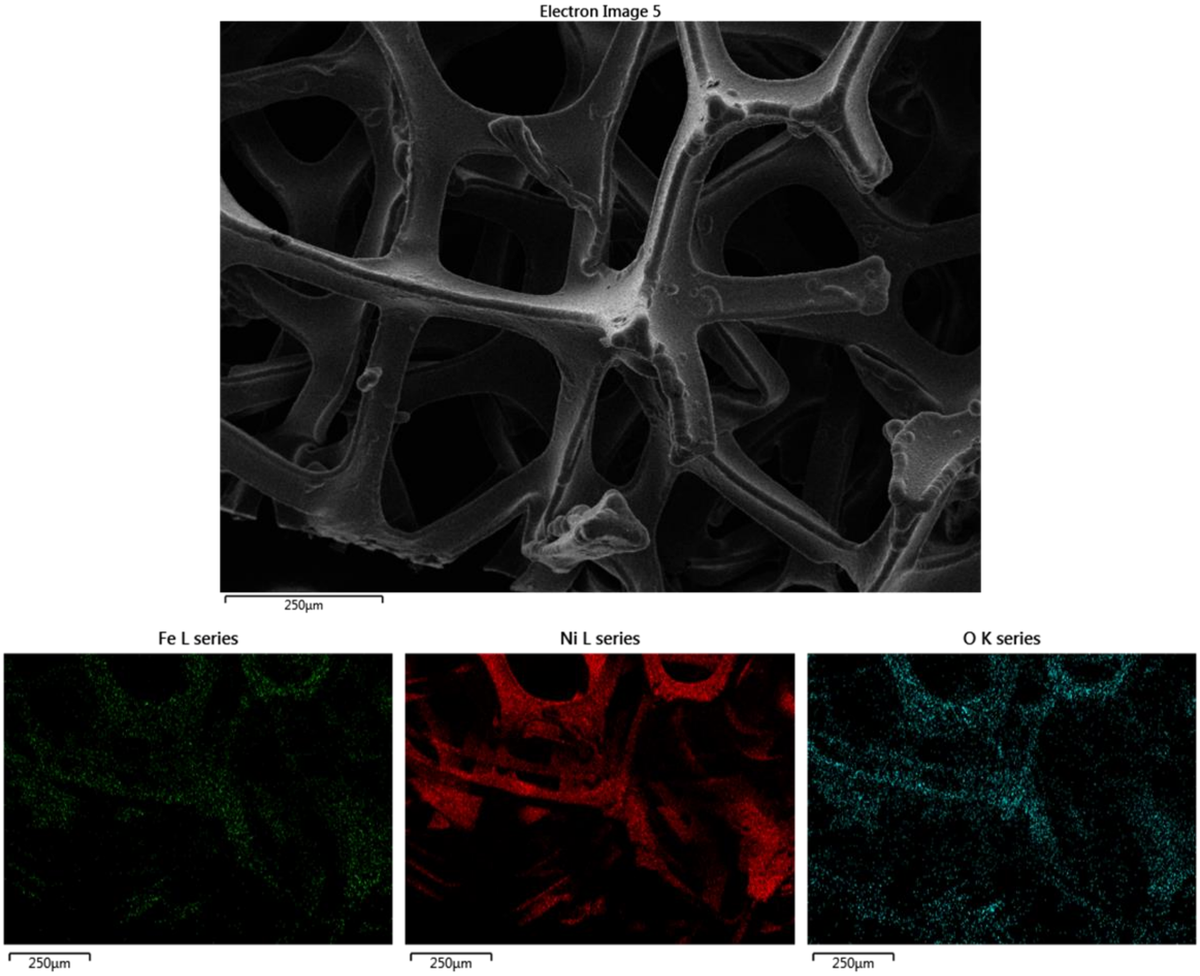

Supplementary Fig. S22. An SEM image and corresponding EDX elemental mapping images of Fe, Ni, and O in *L*-NiFeOOH on Ni foam.

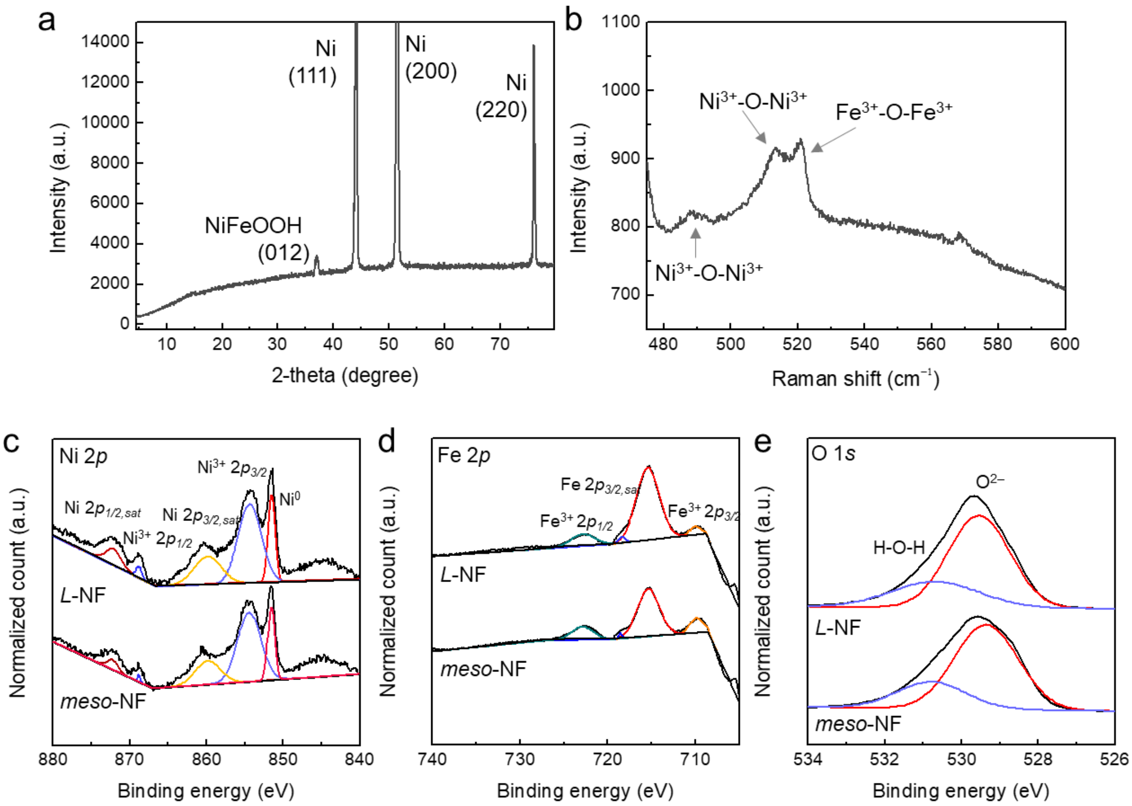

Supplementary Fig. S23. (a) XRD and (b) Raman spectra of *L*-NiFeOOH on Ni foam. And XPS spectra of L-NiFeOOH and meso-NiFeOOH: deconvolution of the (c) Ni 2p, (d) Fe 2p, and (e) O 1s regions.

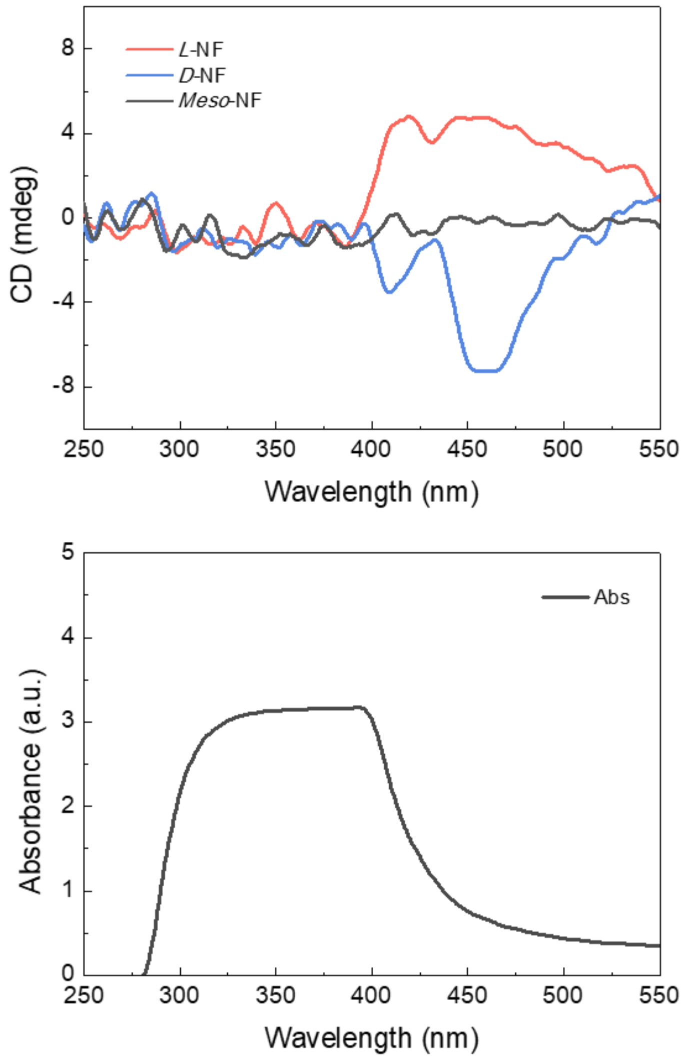

Supplementary Fig. S24. Circular dichroism (CD) and absorbance spectra of the *L-/D-/meso*-NF catalysts on FTO substrates.

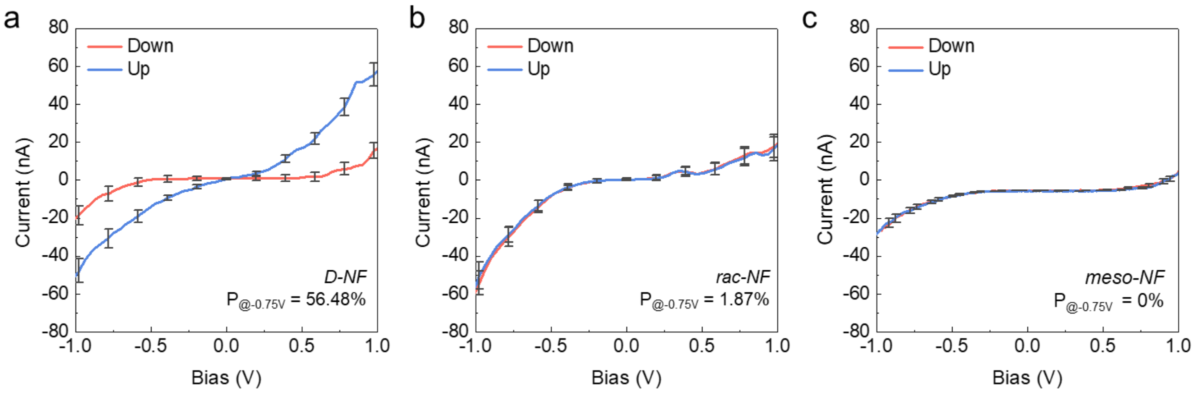

Supplementary Fig. S25. Spin-dependent current behavior depending upon a pre-magnetization direction of tip for (a) *D*-NF, (b) *rac*-NF and (c) *meso*-NF on the Ni foam measured using mCP-AFM in the range from –1.0 to 1.0 V.

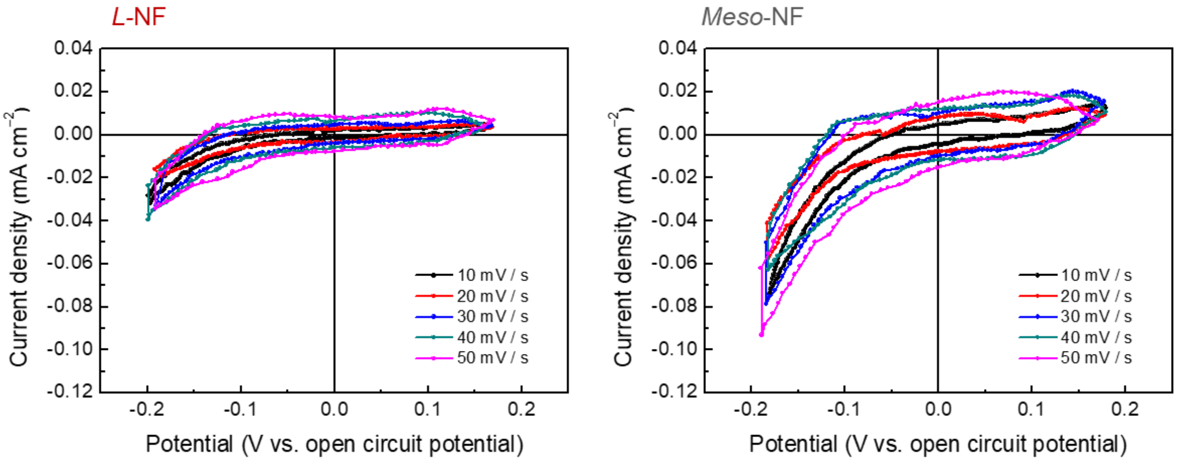

Supplementary Fig. S26. Cyclic voltammetry (CV) analysis of *L*-NF and *meso*-NF at various scan rates ranging from 10 to 50 mV s^{-1} to obtain their electrical double layer capacitance and electrochemically active surface area (ECSA) values.

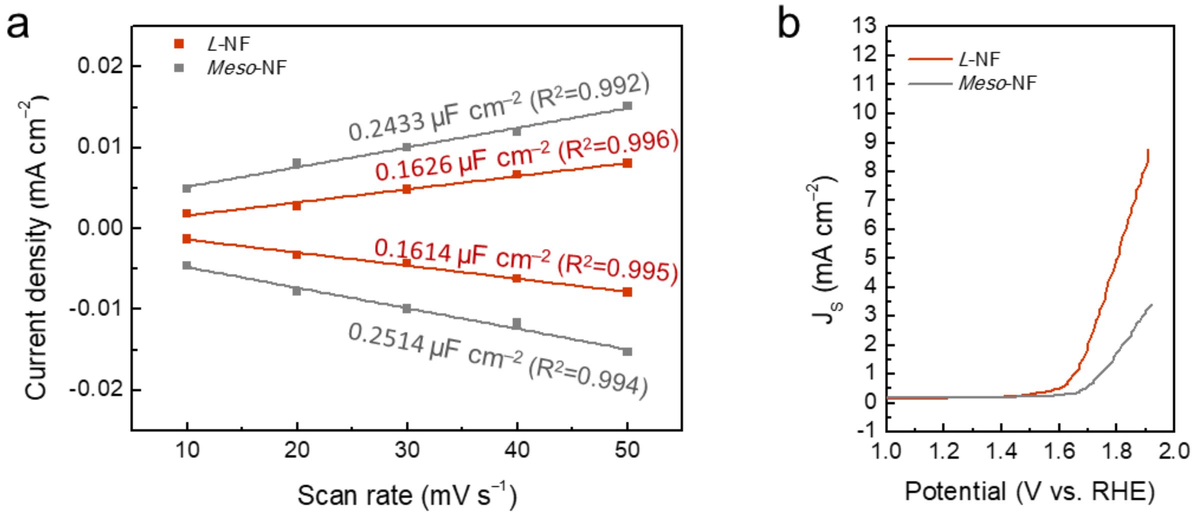

Supplementary Fig. S27. (a) Representative double-layer capacitance measurements for the *L*-NF (dark red) and *meso*-NF (gray) catalysts and (b) LSV curves for both when the current was normalized to the ECSA.

Supplementary Note 3. Determining the specific activity of the NiFeOOH catalysts according to their ECSA values.

To determine the ECSA of the nanomaterials, CV was measured in the non-Faradaic region (−0.19 to +0.18 V vs. open-circuit potential) without stirring the electrolyte at scan rates from 10 to 50 mV s^{-1} (Supplementary Fig. S26). The ECSA is calculated as follows:[R2]

$$ECSA = C_{dl}/C_s, \qquad (S4)$$

where C_{dl} is the electrical double-layer capacitance obtained from the slope of the current vs. scan rate plot (Supplementary Fig. S27a) and C_s is the specific capacitance of the material. A specific capacitance value of 40 μF cm^{-2} was used to calculate the ECSA values of the nanomaterials. Furthermore, the specific activity (J_s) is calculated from the current density per geometric area of the electrode (A) and the ECSA as follows:[R3]

$$J_s = (J_g \text{ x } A)/ECSA, \qquad (S5)$$

where J_g is the current density per unit of the geometric area (A). To determine whether the overpotential reduction in the chiral catalysts arises from differences in the ECSA, Supplementary Fig. S27a shows representative C_{dl} values for *L*-NF and *meso*-NF used to determine their ECSAs. Supplementary Fig. S27b exhibits LSV curves for the *L*-NF (dark red) and *meso*-NF (gray) catalysts after applying a current normalized to the ECSA.

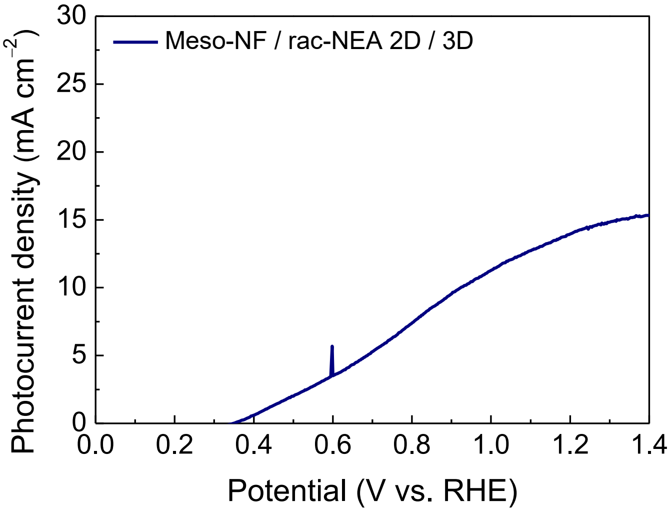

Supplementary Fig. S28. LSV curves of *meso*-NF/*rac*-NEA 2D/3D photoanode.

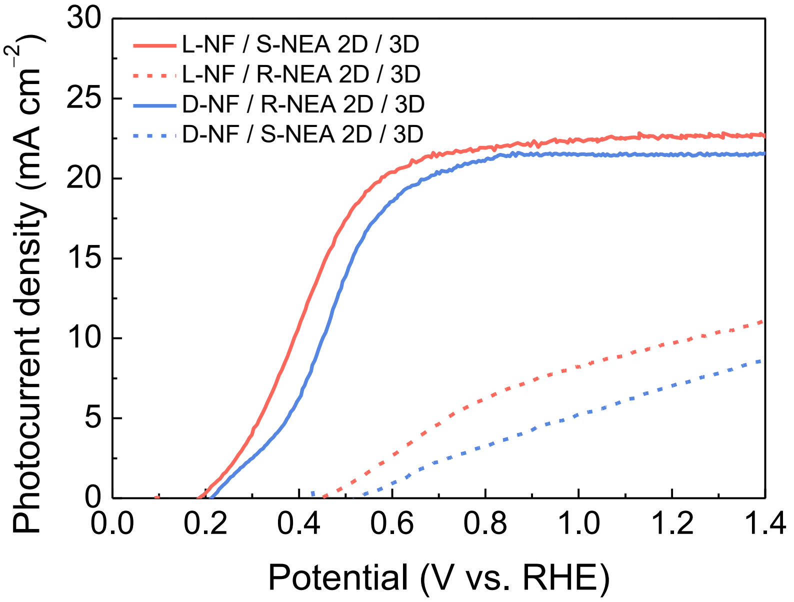

Supplementary Fig. S29. LSV curves of *L*-NF/*S*-NEA 2D/3D (red-solid), *L*-NF/*R*-NEA 2D/3D (red-dotted), *D*-NF/*R*-NEA 2D/3D (blue-solid), and *D*-NF/*S*-NEA 2D/3D (blue-dotted) photoanodes with an area of 0.06 cm^2.

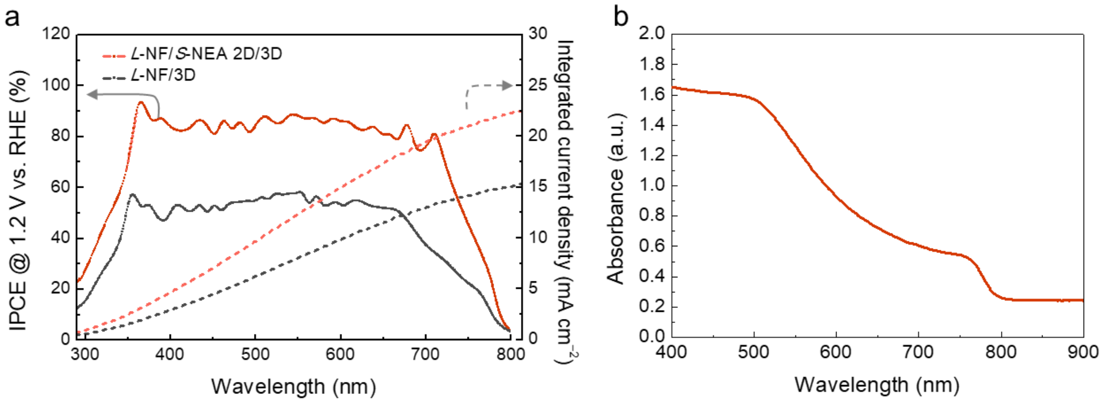

Supplementary Fig. S30. (a) Incident photon-to-current conversion efficiency (IPCE) measurements for the *L*-NF/3D and *L*-NF/*S*-NEA 2D/3D OIHP photoanode in 0.5 M K-Pi (pH 6.5) electrolyte at 1.23 V_{RHE} under 1 sun illumination. The right axis of the IPCE plot shows the integrated current density of the photoanode. (b) A UV-Vis spectrum showing the absorbance by the 3D OIHP on an FTO substrate.

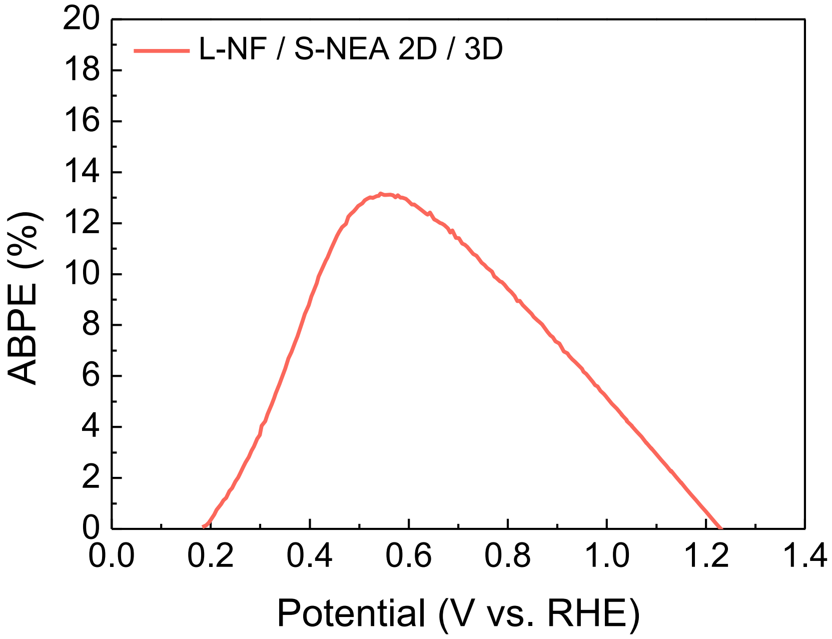

Supplementary Fig. S31. An applied-bias photon-current conversion efficiency (ABPE) plot for the *L*-NF/*S*-NEA 2D/3D OIHP photoanode using the LSV data in Fig. 3d.

Supplementary Table S2. Photoelectrochemical (PEC) performance comparison of the *L*-NF/*S*-NEA 2D/3D OIHP photoanode with previously reported OIHP-based ones.

Water-Splitting System	Photoanode Configuration	Onset Potential (V_{RHE})	Photocurrent Density (@1.23V_{RHE})	ABPE (%)	Electrolyte	Stability	Year	Ref
**PEC-PEC**	**L-NiFeOOH/Ni/Ketjen/S88:P12/S-NEA 2D chiral OIHP/3D OIHP/SnO_2/FTO**	**0.20**	**22.56**	**13.17**	**0.5 M K-Pi (pH6.5)**	**160 h (*j/j₀* ~80%)**	**2023**	**This work**
PEC-PEC	IrO_x/CAB/Au/Spiro-OMeTAD/$(FAPbI_3)_{0.97}(MAPbI_3)_{0.03}$/$SnO_2$/FTO	0.35	24.37	-	0.5 M H_2SO_4 (pH 1.0)	7.6h (j/j_0 ~60%)	2023	R4
PEC-PEC	N foil.3D Ni.NiFe/Au with the addition of CCP/Spiro-OMeTAD/$(CH(NH)_2)_{2(0.93)}(CH_3NH_3)_{(0.07)}PbI_3$ ($FAMAPbI_3$)/SnO_2/ITO	0.28	22.42	8.92	1.0 M KOH (pH 14)	20h (j/j_0 ~60%)	2023	R5
Half-cell PEC	Fe-doped Ni_3S_2/Ni foil/Fusible metal/Au/Poly-TPD/Spiro-OMeTAD/$(FAPbI_3)_{0.95}(MAPbBr_3)_{0.05}$/GTMACI/$SnO_2$/FTO	0.23	23	12.79	1 M NaOH (pH 13)	12h (j/j_0 ~90%)	2023	R6
PV-EC	Ni/GS/Au/Spiro-OMeTAD/$FA_{0.83}Cs_{0.17}Pb(I_{0.8}Br_{0.2})_3$/$SnO_2$/ITO	0.60	17.40	-	1.0 M KOH (pH 14)	12h (j/j_0 ~50%)	2021	R7
Half-cell PEC	Ni.NiFe/Ni foil.3D/$FAMAPbI_3$/SnO_2/$SnCl_2$/ITO	0.56	24.26	9.16	1 M KOH (pH 14)	48h (j/j_0 ~50%)	2021	R8
PV-EC	$FeNi(OH)_x$/Ni/Au/spiro-oMeTAD/$FA_{0.80}MA_{0.15}Cs_{0.05}PbI_{2.55}Br_{0.45}$/$TiO_2$/FTO	0.39	11.6	-	0.5 M NaOH	13h (j/j_0 ~87%)	2021	R9
Half-cell PEC	Ir-WOC/graphite sheet/m-Carbo/$CsPbBr_3$/TiO_2/FTO	0.55	5.1	-	0.1 M KNO_3 (pH 2.5)	2.2h (j/j_0 ~87%)	2019	R10
PV-EC	CC/SC/CC/(5-AVA)$MAPbI_3$/Carbon/ZrO_2/TiO_2/FTO	0.94	12.4	0.85	1 M KOH (pH 14)	12h (j/j_0 ~68%)	2019	R11
PV-EC	Ni/Bi−In−Sn Ingot/Au/spiro-oMeTAD/$MAPbI_3$/TiO_2/FTO	0.52	16	-	1.0 M KOH (pH 14)	5h (j/j_0 ~93%)	2018	R12
PV-EC	Ni/Au/spiro-oMeTAD/$MAPbI_3$/TiO_2/FTO	-	10	-	0.1 M Na_2S (pH 12.8)	100s (j/j_0 ~56%)	2015	R13

j; retention photocurrent density; j_0, initial photocurrent density; PV-EC, photovoltaic-electrochemical; WOC, water oxidation catalyst; CCP, capacitively coupled plasma; ITO, indium tin oxide; poly-TPD, poly(N,N′-*bis*(4-butylphenyl)-N,N′-*bis*(phenyl)benzidine); CAB, conductive adhesive barrier.

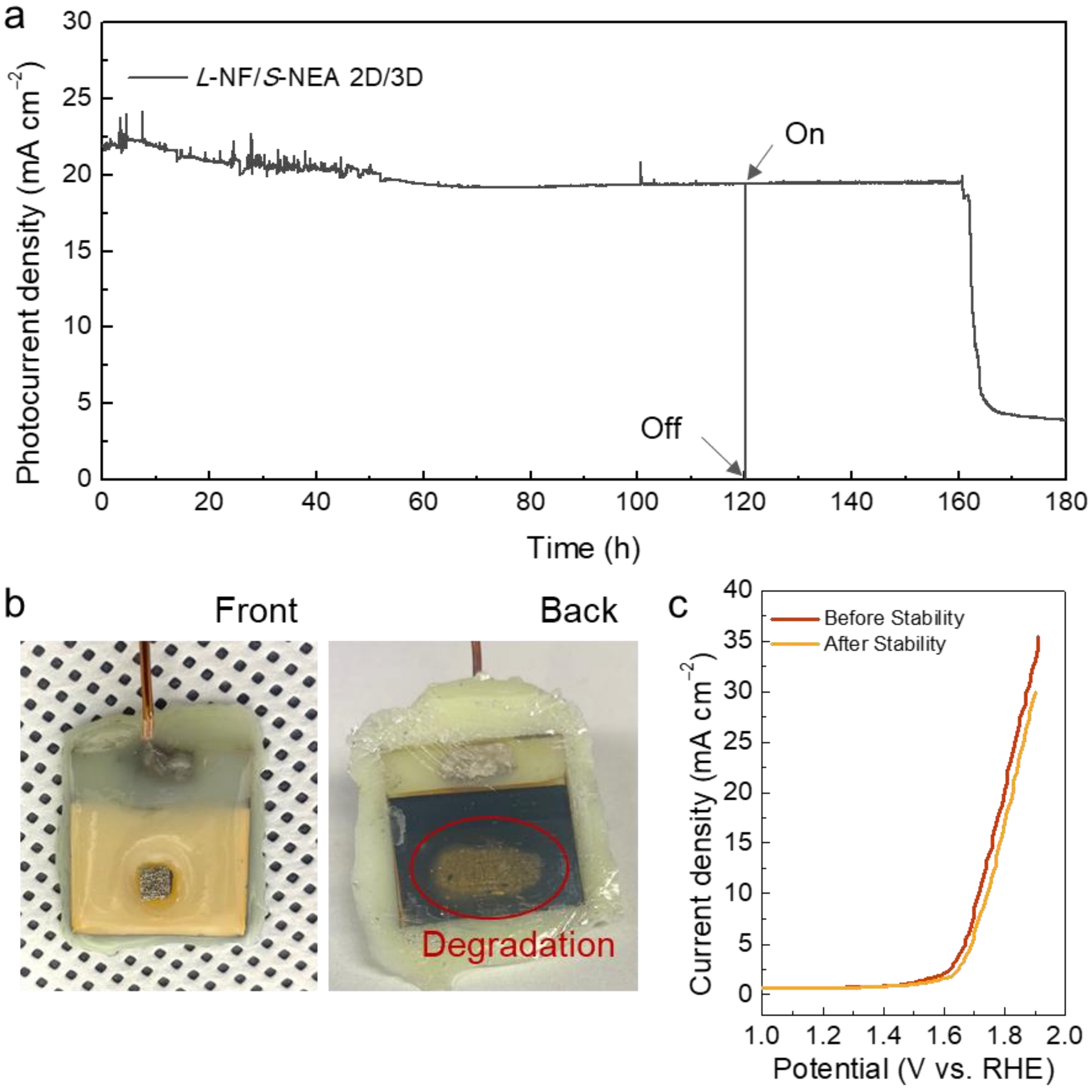

Supplementary Fig. S32. (a) Operational stability of *L*-NF/*S*-NEA 2D/3D OIHP photoanode under continuous operation in 0.5 M K-Pi (pH 6.5) electrolyte at 1.2 V_{RHE} in an ambient atmosphere with an area of 0.06 cm^2. (b) Photographs of front and back of the OIHP photoanode after the stability test. (c) LSV curves of *L*-NF before and after stability test. The voltage was not iR corrected.

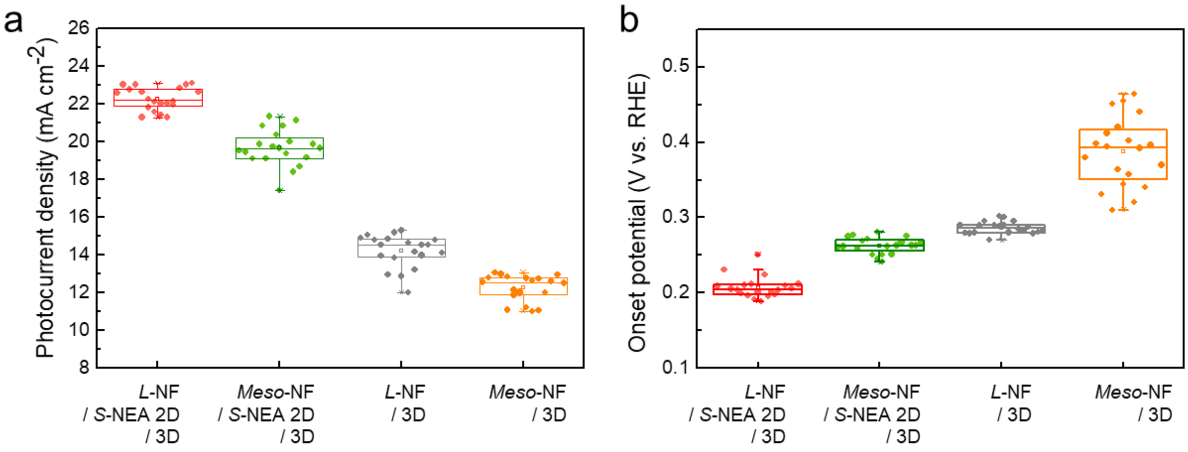

Supplementary Fig. S33. PEC performance statistics of the OIHP-based photoanodes determined from the corresponding J–V curves. (a) Photocurrent density (J_{ph}) and (b) onset potential (V_{onset}) values of the *L*-NF/*S*-NEA 2D/3D (red), *meso*-NF/*S*-NEA 2D/3D (green), *L*-NF/3D (gray), and *meso*-NF/3D (orange) photoanodes.

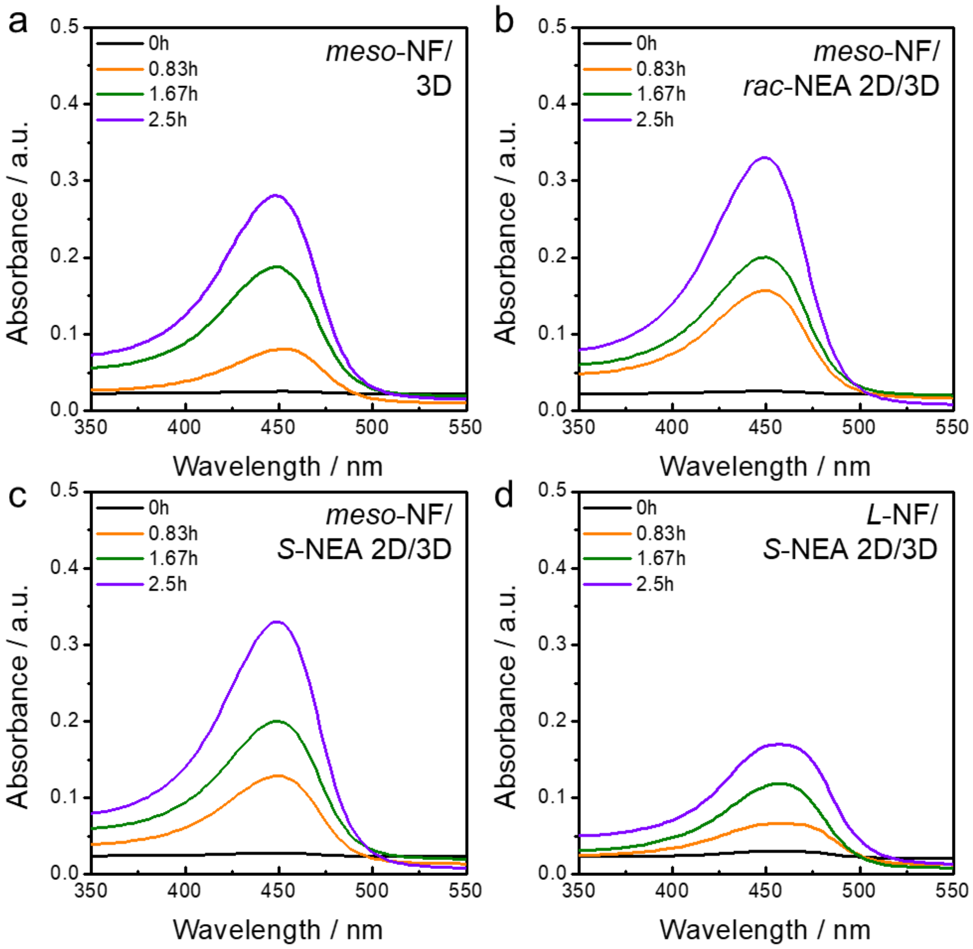

Supplementary Fig. S34. UV-vis absorption spectra from o-toluidine titration of electrolytes (0.5 M Na_2SO_4) as a function of reaction duration for (a) *meso*-NF/3D OIHP, (b) *meso*-NF/*rac*-NEA 2D/3D OIHP, (c) *meso*-NF/*S*-NEA 2D/3D OIHP, and (d) *L*-TA/*S*-NEA 2D/3D OIHP.

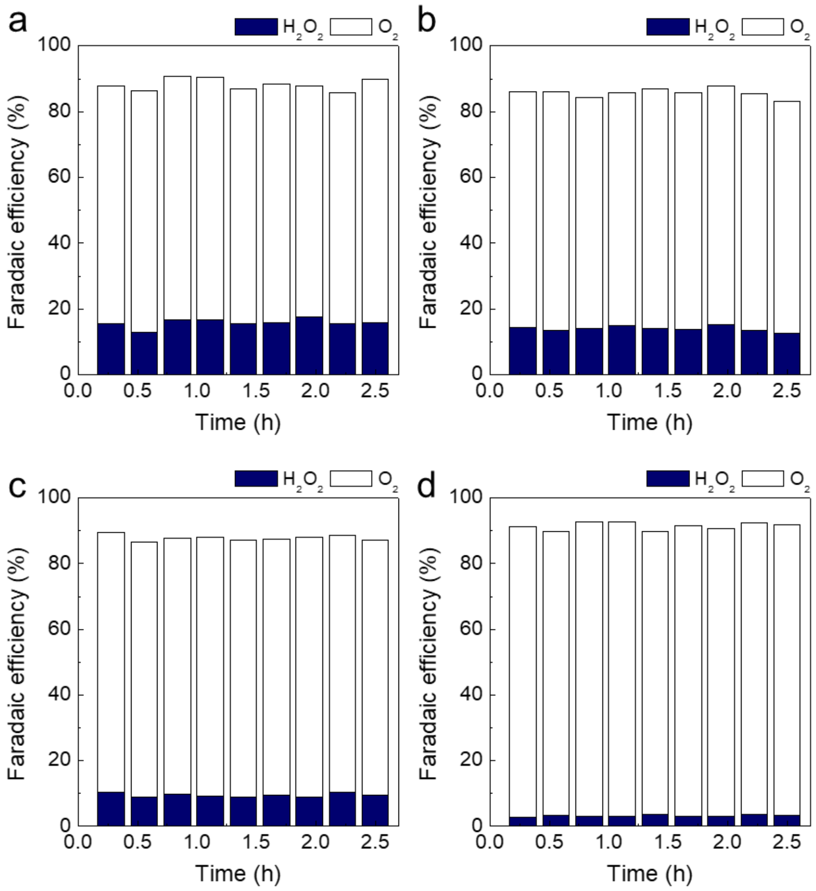

Supplementary Fig. S35. Calculated O_2 and H_2O_2 Faradaic efficiency stacked bar chart for (a) *meso*-NF/3D OIHP, (b) *meso*-NF/*rac*-NEA 2D/3D OIHP, (c) *meso*-NF/*S*-NEA 2D/3D OIHP, and (d) *L*-TA/*S*-NEA 2D/3D OIHP.

Supplementary Note 4. Interpretation of the electrochemical impedance spectroscopy (EIS) analysis results with the equivalent circuit model.

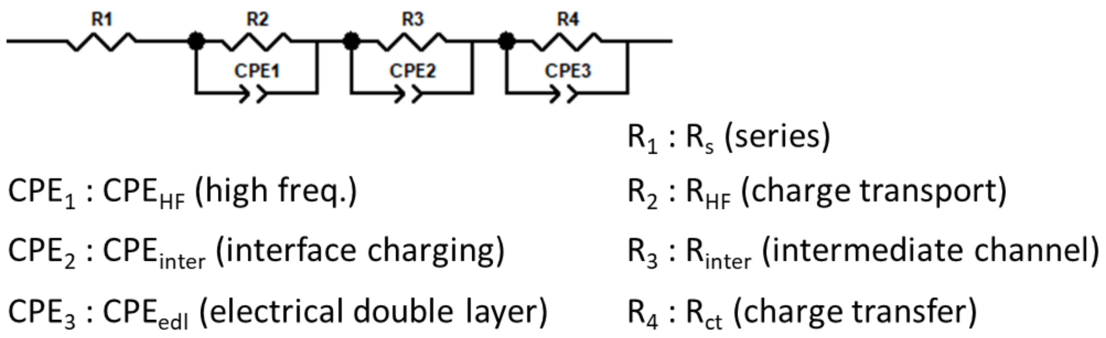

Supplementary Fig. S36. The equivalent circuit using the Voight model to interpret the EIS analysis results.

We quantitatively deduced the PEC parameters from the EIS analysis results by adopting a simple Voight circuit model (Supplementary Fig. S36).[R14] The three deconvoluted semicircles are denoted as follows: high-frequency resistance (R_{HF}) with capacitance (CPE_{HF}), middle-frequency resistance (R_{inter}) with capacitance (CPE_{inter}), and low-frequency resistance (R_{ct}) with capacitance (CPE_{edl}) represented by the characteristic frequencies of 15–50 kHz, 10 Hz, and 0.5–1 Hz, respectively. The fitted impedance data for the PEC water-splitting devices are summarized in Supplementary Table S3. According to the literature on PEC, semicircles in different frequency regions may be assigned to different electrochemical-influencing phenomena. For example, R_{HF} is influenced by the charge transport within stacked semiconductors in a device, R_{inter} reflects the intermediate state at an interface acting as a one-way spin-transport channel while suppressing back-recombination of the charge carriers, and R_{ct} represents the resistance of PEC charge transfer reactions within the electrical double layer.

Supplementary Table S3. Summary of the fitted impedance data for the *L*-NF/3D, *L*-NF/*S*-NEA 2D/3D, and *L*-NF/*rac*-NEA 2D/3D photoanodes.

***L*-NF /3D**	**R_{series} (Ω·cm²)**	**R_{HF} (Ω·cm²)**	**CPE_{HF} ($F·s^{n-1}·cm^{-2}$)**	**R_{inter} (Ω·cm²)**	**CPE_{inter} ($F·s^{n-1}·cm^{-2}$)**	**R_{ct} (Ω·cm²)**	**CPE_{edl} ($F·s^{n-1}·cm^{-2}$)**
0.4 V_{RHE}	3.12	168.9	1.02×10^{-8} (n = 1.03)	-	-	61.24	6.12×10^{-5} (n = 0.91)
0.8 V_{RHE}	3.18	131.2	4.89×10^{-9} (n = 1.02)	-	-	48.91	4.85×10^{-4} (n = 0.82)
1.2 V_{RHE}	3.12	115.3	7.10×10^{-9} (n = 0.99)	-	-	31.02	4.71×10^{-4} (n = 0.83)

***L*-NF /*S*-NEA 2D/3D**	**R_{series} (Ω·cm²)**	**R_{HF} (Ω·cm²)**	**CPE_{HF} ($F·s^{n-1}·cm^{-2}$)**	**R_{inter} (Ω·cm²)**	**CPE_{inter} ($F·s^{n-1}·cm^{-2}$)**	**R_{ct} (Ω·cm²)**	**CPE_{edl} ($F·s^{n-1}·cm^{-2}$)**
0.4 V_{RHE}	2.97	30.51	1.11×10^{-8} (n = 1.09)	6.00	2.01×10^{-6} (n = 0.94)	14.47	5.41×10^{-4} (n = 0.78)
0.8 V_{RHE}	2.27	22.24	1.02×10^{-8} (n = 1.08)	6.96	2.11×10^{-6} (n = 0.95)	6.01	5.28×10^{-4} (n = 0.81)
1.2 V_{RHE}	2.17	19.89	2.01×10^{-8} (n = 1.09)	9.94	1.96×10^{-6} (n = 0.94)	5.01	4.97×10^{-4} (n = 0.78)

***L*-NF /*rac*-NEA 2D/3D**	**R_{series} (Ω·cm²)**	**R_{HF} (Ω·cm²)**	**CPE_{HF} ($F·s^{n-1}·cm^{-2}$)**	**R_{inter} (Ω·cm²)**	**CPE_{inter} ($F·s^{n-1}·cm^{-2}$)**	**R_{ct} (Ω·cm²)**	**CPE_{edl} ($F·s^{n-1}·cm^{-2}$)**
0.4 V_{RHE}	2.12	82.11	9.92×10^{-9} (n = 1.02)	8.01	1.96×10^{-6} (n = 0.93)	72.02	5.97×10^{-4} (n = 0.75)
0.8 V_{RHE}	2.04	70.28	8.57×10^{-9} (n = 1.01)	7.45	1.98×10^{-6} (n = 0.94)	37.21	6.27×10^{-4} (n = 0.71)
1.2 V_{RHE}	1.89	58.9	8.88×10^{-9} (n = 1.02)	6.10	2.01×10^{-6} (n = 0.94)	24.46	7.79×10^{-4} (n = 0.73)

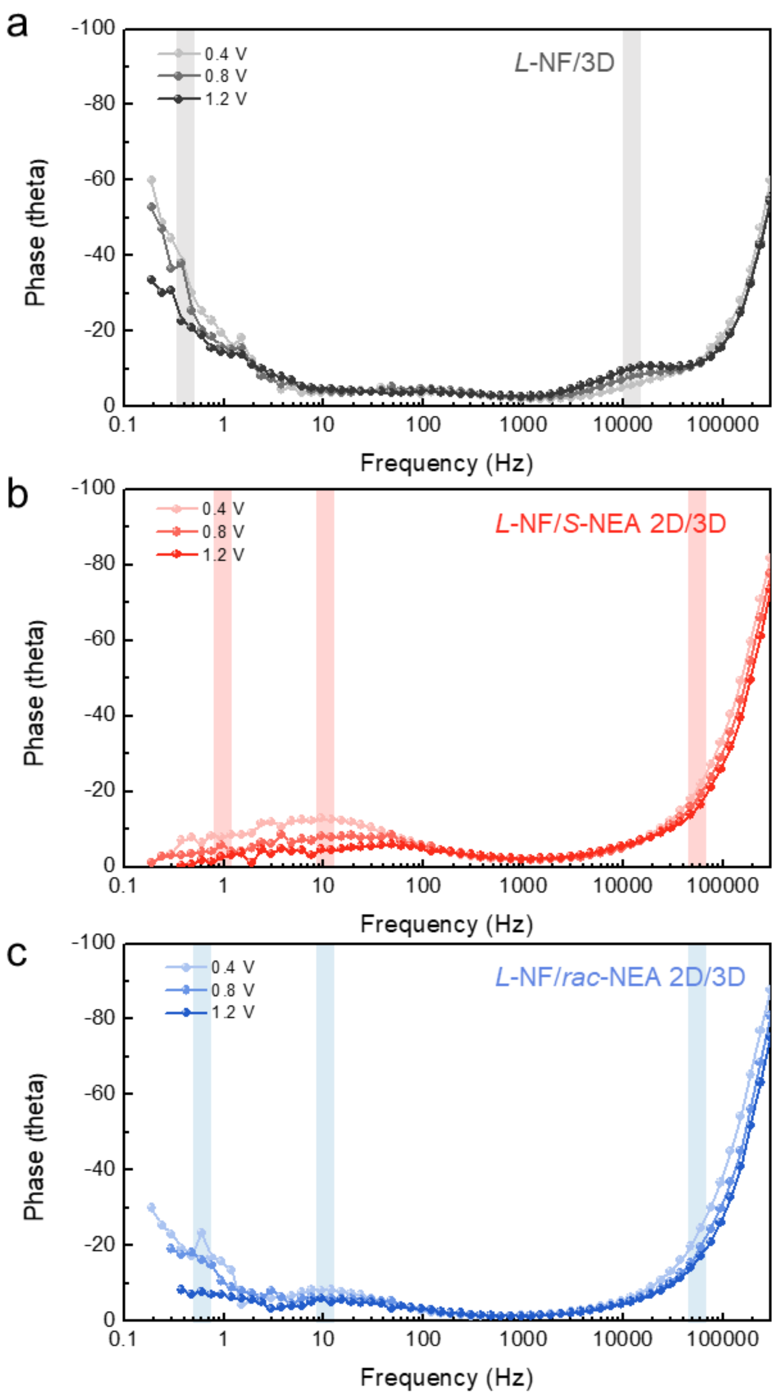

Supplementary Fig. S37. Bode plots of the EIS results for the (a) *L*-NF/3D (black), (b) *L*-NF/*S*-NEA 2D/3D (red), and (c) *L*-NF/*rac*-NEA 2D/3D (blue) photoanodes in 0.5 M K-Pi electrolyte (pH 6.5) under 1 sun illumination.

Supplementary Note 5. Calculation of the rate constants using the IMPS analysis.

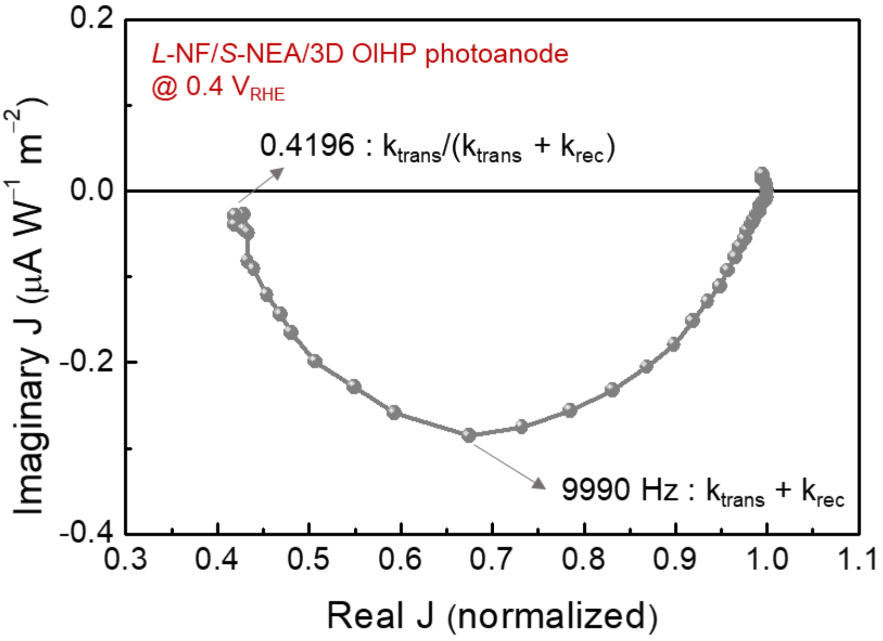

Supplementary Fig. S38. A representative Nyquist plot of the IMPS analysis results for the *L*-NF/*S*-NEA 2D/3D OIHP photoanode at 0.4 V_{RHE}.

The frequency (*f*) at the apex (9990 Hz) is related to the combined rate of charge transport and recombination and the low-frequency intercept (0.4196) indicating the charge-transport efficiency (i.e., k_{trans} / (k_{trans} + k_{rec})); the bulk charge-transport constant (k_{trans}) and the interface recombination rate constant (k_{rec}) are calculated as follows:

$$k_{trans} + k_{rec} = 2\pi f = 2\pi\ (9990) = 62769\ (s^{-1}) \quad (S6)$$

$$k_{trans} = \text{charge-transport efficiency x } (k_{trans} + k_{rec}) = 0.4196 \text{ x } 62769 = 26337.9\ (s^{-1}) \quad (S7)$$

$$k_{rec} = 62769 - 26337.9 = 36431.1\ (s^{-1}) \quad (S8)$$

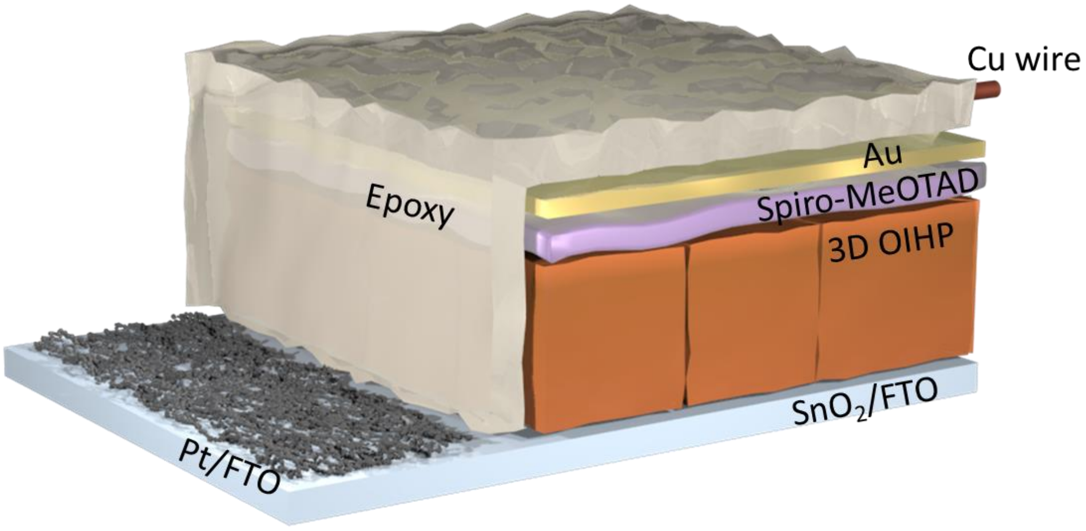

Supplementary Fig. S39. A schematic illustration of an FTO/SnO_2/3D/spiro-MeOTAD/Au photocathode with decoupled Pt/FTO.

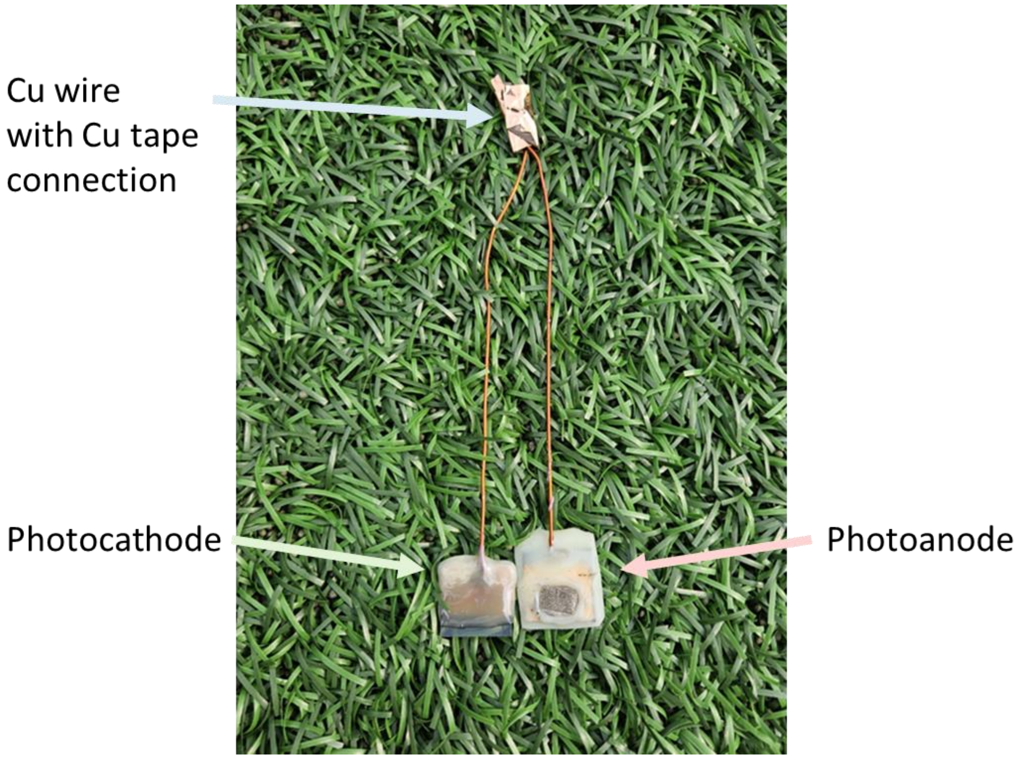

Supplementary Fig. S40. A photograph of the co-planar photocathode-photoanode water-splitting device that can operate under outdoor sunlight. The device had a total active area of 1 cm^2 (the summation of the photocathode area (0.5 cm^2) and photoanode area (0.5 cm^2)).

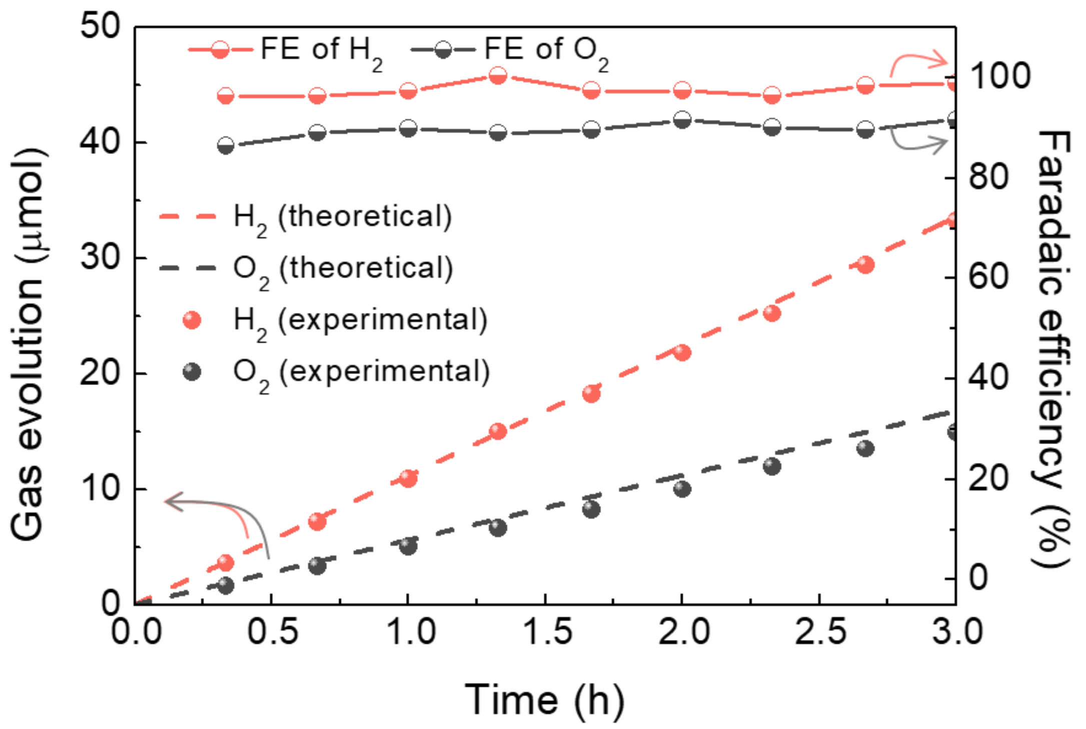

Supplementary Fig. S41. The Faradaic efficiency of the co-planar photocathode-photoanode water-splitting device obtained by comparing the experimental gas chromatography values (spot patterns) with the theoretical values in Fig. 5d (dashed lines) under an unbiased voltage. The active areas were 0.06 cm^2 for the each photoelectrodes

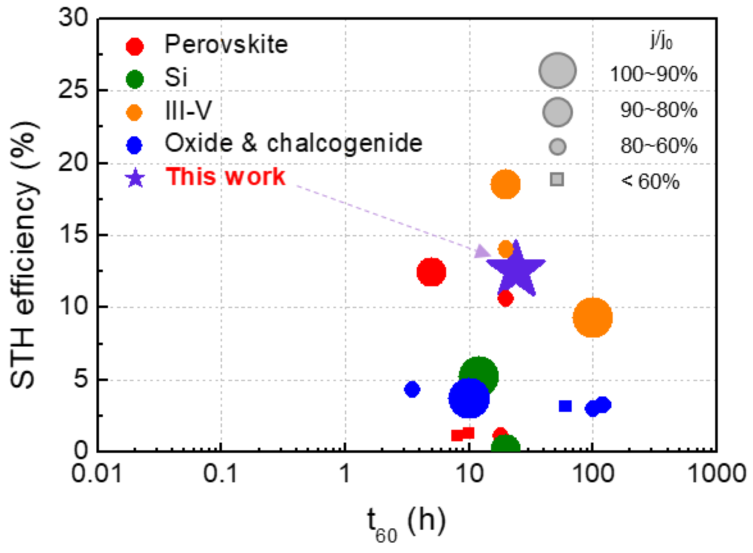

Supplementary Fig. S42. An overall water-splitting performance comparison of previously reported unassisted PEC-PEC systems with our device. The size of the circle corresponds to the STH retention ratio of the devices. *j*, retention photocurrent density; j_0, initial photocurrent density.

Supplementary Table S4. Comparison of the solar-to-hydrogen (STH) efficiency and stability of our PEC-PEC-configured overall water-splitting system with previously reported ones.

Absorber Semiconductor	Photoanode	Photocathode	STH (%)	Stability	Electrolyte	Year	Ref
**Perovskite**	**L-NiFeOOH/Ni/Ketjen/S88:P12/S-NEA 2D chiral OIHP/3D OIHP/SnO_2/FTO**	**Pt/SnO_2/(3D OIHP)/(HTL)/Au**	**12.55**	**24h (j/j0 ~100%)**	**0.5 M K-Pi (pH 6.5)**	**2023**	**This work**
Perovskite	Au/Spiro-OMeTAD/$(FAPbI_3)_{0.97}(MAPbI_3)_{0.03}$/$SnO_2$/FTO	ITO/PTAA/$Cs_{0.05}FA_{0.85}MA_{0.1}Pb(I_{0.95}Br_{0.05})_3$/LiF/$C_{60}$/BCP/Ag	12.4	5h (j/j_0 ~80%)	0.5 M H_2SO_4 (pH 1.0)	2023	R4
Perovskite	Nickel foil.3D Ni.NiFe/Au with the addition of CCP/Spiro- OMeTAD/ $(CH(NH)_2)_{2(0.93)}(CH_3NH_3)_{(0.07)}PbI_3$ $(FAMAPbI_3)$/SnO_2/ITO	ITO/PTAA/polymer/$(FAPbI_3)_{0.85}(MAPbBr)_{0.15}$(FAMA)/$PC_{60}$BM/BCP/Au/CCP/NiPCoP/Ni foil	10.6	20h (j/j_0 ~60%)	1.0 M KOH (pH 14)	2023	R5
Perovskite	Pt/Ti/n^+-Si/n-Si/p^+-Si/Ag	ITO/NiOx/PTAA/halide perovskite /PEIE/C60/ITO/TiO2/Pt	3.6	8h (j/j_0 ~50%)	1.0 M K-Bi (pH 9.0)	2023	R15
Si	NiCOFe-B_i/CPF-TCB/Sb_2S_3	Si/TiO2/Pt	5.2	12h (j/j_0 ~95%)	1.0 M phosphate-buffered saline (pH6.8)	2022	R16
Perovskite	TiCo/$BiVO_4$/FTO	FTO/NiOx/PTAA/$FAMA_{0.22}Pb_{1.32}I_{3.2}Br_{0.66}$/PCBM/GE/Pt	1.3	10h (j/j_0 ~30%)	0.1 M K-Bi / 0.1 M K_2SO_4 (pH 8.5)	2021	R17
Oxide & Chalcogenide	NiFeOx-Bi/$BiVO_4$	CZTS/HfO_2/CdS/HfO_2-Pt	3.17	60h (j/j_0 ~50%)	0.2 M K-Pi (pH 7.0)	2021	R18
Oxide & Chalcogenide	$NiFeO_x$/CTF-BTh/Mo:$BiVO_4$	Cu_2O/CTF-BTh/MoS_x	3.24	120h (j/j_0 ~60%)	0.5 M K-Bi (pH 9.0)	2021	R19
Oxide & Chalcogenide	NiOOH/FeOOH/$BiVO_4$	Si/TiO2/Pt	3.7	10h (j/j_0 ~100%)	1.0 M KOH / 1.0M $HClO_4$	2021	R20
Oxide & Chalcogenide	Co_4O_4/pGO/$BiVO_4$/SnOx	CuO_x/PIP/TiO_x/Pt	4.3	3.5h (j/j_0 ~60%)	0.5 M K-Pi (pH 7.0)	2021	R21
Perovskite	TiCo/$BiVO_4$/FTO	FTO/NiOx/$FAMA_{0.22}Pb_{1.1}I_{3.2}Br_{0.66}$/PCBM/PEIE/Ag/FM/Ti foil/TiO_2/H_2ase	1.1	8h (j/j_0 ~20%)	50 mM ES (pH 6.0)	2019	R22
Perovskite	TiCo/$BiVO_4$/FTO	FTO/NiOx/$FAMA_{0.22}Pb_{1.1}I_{3.2}Br_{0.66}$ /PCBM/PEIE/Au/Pt	1.1	18h (j/j_0 ~60%)	0.1 M K-Bi (pH 8.5)	2018	R23
III-V	RuO_x/GaAs/GaInAs	GaInP/AlInP/$AlInPO_x$/TiO_2/Rh	18.5	20h (j/j_0 ~80%)	0.5 M K-Pi (pH 7.0)	2018	R24
Oxide	$NiFeO_x$/Mo:$BiVO_4$	Cu_2O/Ga_2O_3/TiO_2/RuO_2	3	100h (j/j_0	0.2 M K-Bi	2018	R25

& Chalcogenide				~60%)	(pH 8.5)		
III-V	Ti mesh/CoP/InGaP/GaAs	GaAs/InGaP/TiO_2/Ni	9.3	100h (j/j_0 ~90%)	1.0 M K-Bi (pH 9.3)/ 1.0 M H_2SO_4 (pH 0)	2016	R26
Si	WO_3/FTO/p^+-Si/n-Si/n^+-Si/$TiSi_2$	$PtSi_2$/p^+-Si/p-Si/n^+- Sin/Ti/TiO_2/Pt	0.2	20h (j/j_0 ~80%)	1.0 M $HClO_4$	2015	R27
III-V	RuO_2/Ge/GaInAs	GaInP/$Al_xIn_{1-2}P$/$AlInPO_x$/Rh	14	20h (j/j_0 ~60%)	1.0 M $HClO_4$	2015	R28

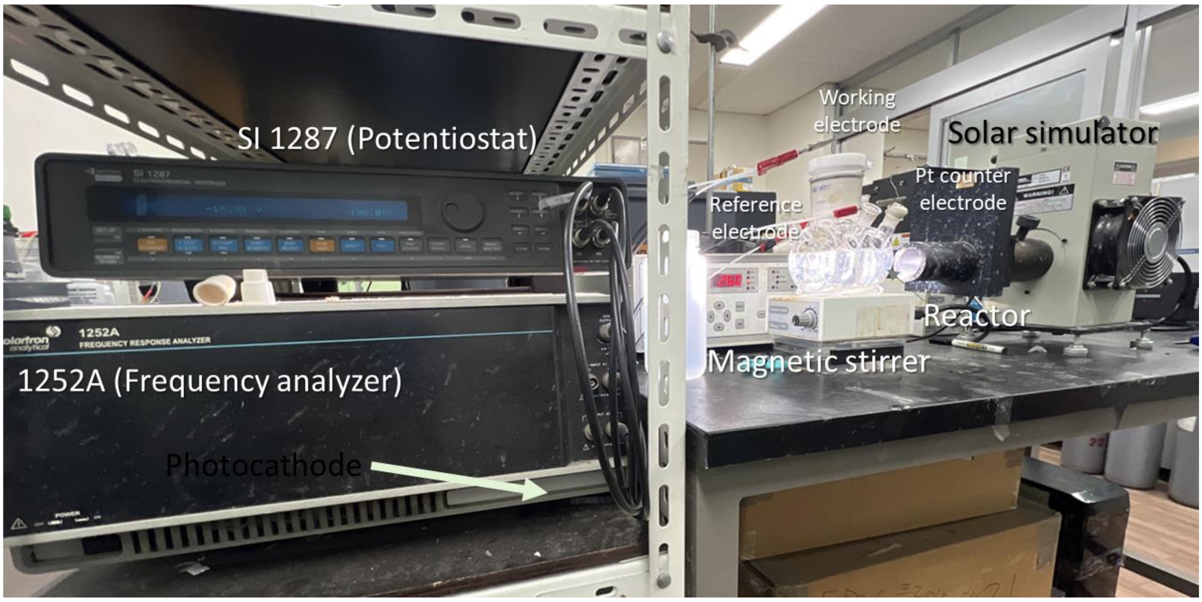

Supplementary Fig. S43. The photographs of experimental set-up for the photoelectrochemical/electrochemical cells. A potentiostat (SI 1287, Solartron, UK) and a frequency analyzer (1252A, Solartron, Leicester, UK) were used to analysis for the cyclic voltammetry, impedance measurements.

Supplementary References

R1 Lu, H. P., Wang, J. Y., Xiao, C. X., Pan, X., Chen, X. H., Brunecky, R., Berry, J. J., Zhu, K., Beard, M. C. & Vardeny, Z. V. Spin-dependent charge transport through 2D chiral hybrid lead-iodide perovskites. *Sci. Adv.* **5**, eaay0571 (2019).

R2 Vadakkayil, A., Clever, C., Kunzler, K. N., Tan, S. S., Bloom, B. P. & Waldeck, D. H. Chiral electrocatalysts eclipse water splitting metrics through spin control. *Nat. Commun.* **14**, 1067 (2023).

R3 Park, Y. S., Park, Y. Sun., Jang, G., Sohn, I., Lee, H., Tan, J., Juwon, Y., Ma, S., Lee, J., Lee, C., Moon, S., Im, H., Chung, S. M., Yu, S., Kim, H. & Moon, J. Efficient solar fuel production enabled by an iodide oxidation reaction on atomic layer deposited MoS_2. *Carbon Energy* e366 (2023).

R4 Fehr, A. M. K., Agrawal, A., Mandani, F., Conrad, C. L., Jiang, Q., Park, S. Y., Alley, O., Li, B., Sidhik, S., Metcalf, I., Botello, C., Young, J. L., Even, J., Blancon, J. C., Deutsch, T. G., Zhu. K., Albrecht, S., Toma, F. M., Wong, M. & Mohite, A. D. Integrated halide perovskite photoelectrochemical cells with solar-driven water-splitting efficiency of 20.8%. *Nat. Commun.* **14**, 3797 (2023).

R5 Rhee, R., Kim, T. G., Jang, G. Y., Bae, G., Lee, J. H., Lee, S., Kim, S., Jeon, S. & Park, J. H. Unassisted overall water splitting with a solar-to-hydrogen efficiency of over 10% by coupled lead halide perovskite photoelectrodes. *Carbon Energy* **5**, 1 (2023).

R6 Choi, H., Kim, Y.Y., Seo, S., Jung, Y., Yoo, S. M., Moon, C.S., Jeon, N.J., Lee, S., Lee, K., Toma, F.M., Seo, J. & Lee, S. Suppression of undesired losses in organometal halide perovskite-based photoanodes for efficient photoelectrochemical water splitting. *Adv. Energy Mater.* **13**, 2300951 (2023).

R7 Wang, M. J., Li, Y. C., Cui, X. H., Zhang, Q. X., Pan, S. J., Mazumdar, S., Zhao, Y. & Zhang, X. D. High-performance and stable perovskite-based photoanode encapsulated by blanket-cover method. *ACS Appl. Energy Mater.* **4**, 7526–7534 (2021).

R8 Kim, T. G., Lee, J. H., Hyun, G., Kim, S., Chun, D. H., Lee, S., Bae, G., Oh, H. S., Jeon, S. & Park, J. H. Monolithic lead halide perovskite photoelectrochemical cell with 9.16% applied bias photon-to-current efficiency. *ACS Energy Lett.* **7**, 320–327 (2022).

R9 Chen, H. J., Zhang, M., Tran-Phu, T., Bo, R., Shi, L., Di, I., Bing, J., Pan, J., Singh, S., Lipton-Duffin, J., Wu, T., Amal, R., Huang, S., Ho-Baillie, A. W. Y. & Tricoli, A. Integrating low-cost earth-abundant co-catalysts with encapsulated perovskite solar cells for efficient and stable overall solar water splitting. *Adv. Funct. Mater.* **31**, 2008245 (2021).

R10 Poli, I., Hintermair, U., Regue, M., Kumar, S., Sackville, E. V., Baker, J., Watson, T. M., Eslava, S. & Cameron, P. J. Graphite-protected $CsPbBr_3$ perovskite photoanodes functionalised with water oxidation catalyst for oxygen evolution in water. *Nat. Commun.* **10**, 2097 (2019).

R11 Tao, R., Sun, Z. X., Li, F. Y., Fang, W. C. & Xu, L. Achieving organic metal halide perovskite into a conventional photoelectrode: outstanding stability in aqueous solution and high-efficient photoelectrochemical water splitting. *ACS Appl. Energy Mater.* **2**, 1969–1976 (2019).

R12 Nam, S., Mai, C. T. K. & Oh, I. Ultrastable Photoelectrodes for Solar Water Splitting Based on Organic Metal Halide Perovskite Fabricated by Lift-Off Process. *ACS Appl. Mater. Interfaces* **10**, 14659–14664 (2018).

R13 Da, P. M., Cha, M. Y., Sun, L., Wu, Y. Z., Wang, Z. S. & Zheng, G. F. High-performance perovskite photoanode enabled by ni passivation and catalysis. *Nano Lett.* **15**, 3452–3457 (2015).

R14 Yang, W., Moehl, T., Service, E. & Tilley, S. D. Operando Analysis of Semiconductor Junctions in Multi-Layered Photocathodes for Solar Water Splitting by Impedance Spectroscopy. *Adv. Energy Mater.* **11**, 2003569 (2021)

R15 Moon, C., Martinho, F. M. A., Jung, G. H., Koh, J., Assar, A., Nam, S. W., Canulescu, S. & Shin, B. Dual-purpose tunnel oxide passivated contact on silicon photoelectrodes with high photovoltages for tandem photoelectrochemical devices enabling unassisted water splitting. *J. Mater. Chem. A* **11**, 4194–4204 (2023).

R16 Wang, L., Lian, W. T., Liu, B., Lv, H. F., Zhang, Y., Wu, X. J., Wang, T., Gong, J. L., Chen, T. & Xu, H. X. A transparent, high-performance, and stable Sb_2S_3 photoanode enabled by heterojunction engineering with conjugated polycarbazole frameworks for unbiased photoelectrochemical overall water splitting devices. *Adv. Mater.* **34**, 2200723 (2022).

R17 Pornrungroj, C., Andrei, V., Rahaman, M., Uswachoke, C., Joyce, H. J., Wright, D. S. & Reisner, E. Bifunctional perovskite-$BiVO_4$ tandem devices for uninterrupted solar and electrocatalytic water splitting cycles. *Adv. Funct. Mater.* **31**, 2008182 (2021).

R18 Huang, D. W., Wang, K., Li, L. T., Feng, K., An, N., Ikeda, S., Kuang, Y. B., Ng, Y. & Jiang, F. 3.17% efficient Cu_2ZnSnS_4-$BiVO_4$ integrated tandem cell for standalone overall solar water splitting. *Energy Environ. Sci.* **14**, 1480–1489 (2021).

R19 Zhang, Y., Lv, H. F., Zhang, Z., Wang, L., Wu, X. J. & Xu, H. X. Boosting the performance of Cu_2O photocathodes for unassisted solar water splitting devices. *Nat. Catal.* **1**, 412–420 (2018).

R20 Liu, B., Wang, S. J., Feng, S. J., Li, H., Yang, L. F., Wang, T. & Gong, J. L. Double-side Si photoelectrode enabled by chemical passivation for photoelectrochemical hydrogen and oxygen evolution reactions. *Adv. Funct. Mater.* **31**, 2007222 (2021).

R21 Ye, S., Shi, W., Liu, Y., Li, D., Yin, H., Chi, H., Luo, Y., Ta, N., Fan, F., Wang, X. & Li C. Unassisted photoelectrochemical cell with multimediator modulation for solar water splitting exceeding 4% solar-to-hydrogen efficiency. *J. Am. Chem. Soc.* **14**, 12499–12508 (2021).

R22 Moore, E. E., Andrei, V., Zacarias, S., Pereira, I. A. C. & Reisner, E. Integration of a hydrogenase in a lead halide perovskite photoelectrode for tandem solar water splitting. *ACS Energy Lett.* **5**, 232–237 (2020).

R23 Andrei, V., Hoye, R. L. Z., Crespo-Quesada, M., Bajada, M., Ahmad, S., De Volder, M., Friend, R. & Reisner, E. Scalable triple cation mixed halide perovskite-$BiVO_4$ tandems for bias-free water splitting. *Adv. Energy Mater.* **8**, 1801403 (2018).

R24 Cheng, W. H., Richter, M. H., May, M. M., Ohlmann, J., Lackner, D., Dimroth, F., Hannappel, T., Atwater, H. A. & Lewerenz, H. J. Monolithic photoelectrochemical device for direct water splitting with 19% efficiency. *ACS Energy Lett.* **3**, 1795–1800 (2018).

R25 Pan, L. F., Kim, J. H., Mayer, M. T., Son, M. K., Ummadisingu, A., Lee, J. S., Hagfeldt, A., Luo, J. S. & Gratzel, M. Boosting the performance of Cu_2O photocathodes for unassisted solar water splitting devices. *Nat. Catal.* **1**, 412–420 (2018).

R26 Sun, K., Liu, R., Chen, Y. K., Verlage, E., Lewis, N. S. & Xiang, C. X. A stabilized, intrinsically safe, 10% efficient, solar-driven water-splitting cell incorporating earth-

abundant electrocatalysts with steady-state pH gradients and product separation enabled by a bipolar membrane. *Adv. Energy Mater.* **6**, 1600379 (2016).

R27 Walczak, K., Chen, Y., Karp, C., Beeman, J. W., Shaner, M., Spurgeon, J., Sharp, I. D., Amashukeli, X., West, W., Jin, J., Lewis, N. S. & Xiang, C. Modeling, simulation, and fabrication of a fully integrated, acid-stable, scalable solar-driven water-splitting system. *ChemSusChem* **8**, 544–551. (2015).

R28 May, M. M., Lewerenz, H. J., Lackner, D., Dimroth, F. & Hannappel, T. Efficient direct solar-to-hydrogen conversion by in situ interface transformation of a tandem structure. *Nat. Commun.* **6**, 8286 (2015).
