# Supplementary material for: A dual spin-controlled chiral two-/three-dimensional perovskite artificial leaf for efficient overall photoelectrochemical water splitting
Source: Nat Commun. 2024 Jun 1;15:4672. doi: 10.1038/s41467-024-49216-x (PMC11144254; doi:10.1038/s41467-024-49216-x)
Supplement: Supplementary file 3 — Description of Additional Supplementary Files [file 41467_2024_49216_MOESM3_ESM.pdf]

Description of Additional Supplementary files

Supplementary Movie 1. Solar water splitting using the co-planar OIHP-based photocathode–photoanode system under outdoor condition.
